# Blockade of C5aR1 resets M1 via gut microbiota-mediated PFKM stabilization in a TLR5-dependent manner

**DOI:** 10.1038/s41419-024-06500-4

**Published:** 2024-02-08

**Authors:** Jie Zhao, Chen Yao, Yongqin Qin, Hanyong Zhu, Hui Guo, Binbin Ji, Xueqin Li, Na Sun, Rongqing Li, Yuzhang Wu, Kuiyang Zheng, Yuchen Pan, Tingting Zhao, Jing Yang

**Affiliations:** 1 Jiangsu International Laboratory of Immunity and Metabolism, Jiangsu Province Key Laboratory of Immunity and Metabolism, Department of Pathogenic Biology and Immunology, Xuzhou Medical University, Xuzhou, Jiangsu China; 2 Department of Immunology, Medical College, Yangzhou University, Yangzhou, China; 3 National Experimental Demonstration Center for Basic Medicine Education, Xuzhou Medical University, Xuzhou, Jiangsu China; 4 Chongqing International Institute for Immunology, Chongqing, China

**Keywords:** Cancer immunotherapy, Immunotherapy

## Abstract

Targeting C5aR1 modulates the function of infiltrated immune cells including tumor-associated macrophages (TAMs). The gut microbiome plays a pivotal role in colorectal cancer (CRC) tumorigenesis and development through TAM education. However, whether and how the gut flora is involved in C5aR1 inhibition-mediated TAMs remains unclear. Therefore, in this study, genetic deletion of *C5ar1* or pharmacological inhibition of C5aR1 with anti-C5aR1 Ab or PMX-53 in the presence or absence of deletion Abs were utilized to verify if and how C5aR1 inhibition regulated TAMs polarization via affecting gut microbiota composition. We found that the therapeutic effects of C5aR1 inhibition on CRC benefited from programming of TAMs toward M1 polarization via driving AKT2-mediated 6-phosphofructokinase muscle type (PFKM) stabilization in a TLR5-dependent manner. Of note, in the further study, we found that C5aR1 inhibition elevated the concentration of serum IL-22 and the mRNA levels of its downstream target genes encoded antimicrobial peptides (AMPs), leading to gut microbiota modulation and flagellin releasement, which contributed to M1 polarization. Our data revealed that high levels of C5aR1 in TAMs predicted poor prognosis. In summary, our study suggested that C5aR1 inhibition reduced CRC growth via resetting M1 by AKT2 activation-mediated PFKM stabilization in a TLR5-dependent manner, which relied on IL-22-regulated gut flora.

## Introduction

Colorectal cancer (CRC) is the third most common cancer [1]. Although early diagnosis and personalized medicine have developed rapidly in the last decade, CRC has become a global medical burden owing to its high morbidity [2, 3].

Increased researches show that complement, the codominant central mediator of humoral immunity, is a critical factor in tumor initiation, development, and chemotherapy resistance [4]. Among them, intratumoral complement 5a (C5a) is increased in patients with CRC after radiotherapy [5], which can suppress cytotoxic T-cell function by recruiting myeloid-derived suppressor cells (MDSCs) to the tumor microenvironment [6], thereby augmenting inflammation-mediated intestinal tumorigenesis [7]. By binding to its receptor C5aR1, C5a promotes the invasion and metastasis of CRC in vitro and in vivo [8, 9]. In addition, the C5a/C5aR1 axis is also critical for β-catenin stabilization, which is required for the maintenance of stemness in colorectal cancer stem cells [9]. In contrast, pharmacological inhibition of C5aR1 signaling by its antagonist PMX-205 or deletion of C5aR1 is sufficient to attenuate the immunosuppressive microenvironment, suppress tumor growth, and reduce lung metastases [10, 11], highlighting the potential value of targeting C5aR1 for CRC treatment.

TAMs can be reset to M1 polarization by regulating their metabolism [12]. It has been shown that in macrophages, the specific metabolic pathways are closely associated with their phenotype and function. Additionally, in the presence of glucose, M1 polarization relies on glycolysis as a source. 6-phosphofructokinase, muscle type (PFKM), is one of the key rate-limiting enzymes in glycolysis. PFKM can induce glycolysis in hepatocellular carcinoma cells [13], whereas inhibition of PFKM can dampen glycolysis in macrophages [14], affecting macrophage functions and tumor development.

A large number of studies have demonstrated that the gut microbiota is involved in mediating several steps of CRC via influencing the role of infiltrated immune cells and augmenting the therapeutic efficacy of anticancer drugs. *Fusobacterium nucleatum* (*F.nucleatum*) drives colorectal carcinogenesis by activating β-catenin signaling and Toll-like receptor 4 (TLR4) and is negatively associated with the prognosis of CRC [15, 16]. *Peptostreptococcus anaerobius* enriches in the fecal and mucosal microbiota from CRC patients, and promotes tumorigenesis [17]. Moreover, trimethylamine N-oxide, a metabolite of the gut microbiota, drives M1 polarization [18].

In the present study, we investigated the phenotype and functions of TAMs and determined the composition and roles of gut microbiota in *C5ar1* knockout (*C5ar1*^*-/-*^) mice bearing CRC tumors. We also clarified the underlying mechanisms involved in the gut-microbiota/metabolite-modulated M1 phenotype in the presence of C5aR1 deletion or inhibition in a CRC mouse model. Our data demonstrated that deletion or pharmaceutical inhibition of C5aR1 contributed to M1 polarization via TLR5/AKT2-mediated PFKM stabilization, which could depend on IL-22-mediated releasement of bacterial flagellin. Our data suggest that inhibition of C5aR1 and adjuvants of the gut microbiota could have great potential for CRC treatment.

## Results

### C5aR1 inhibition re-programs TAMs toward M1 phenotype

**Fig. 1:**
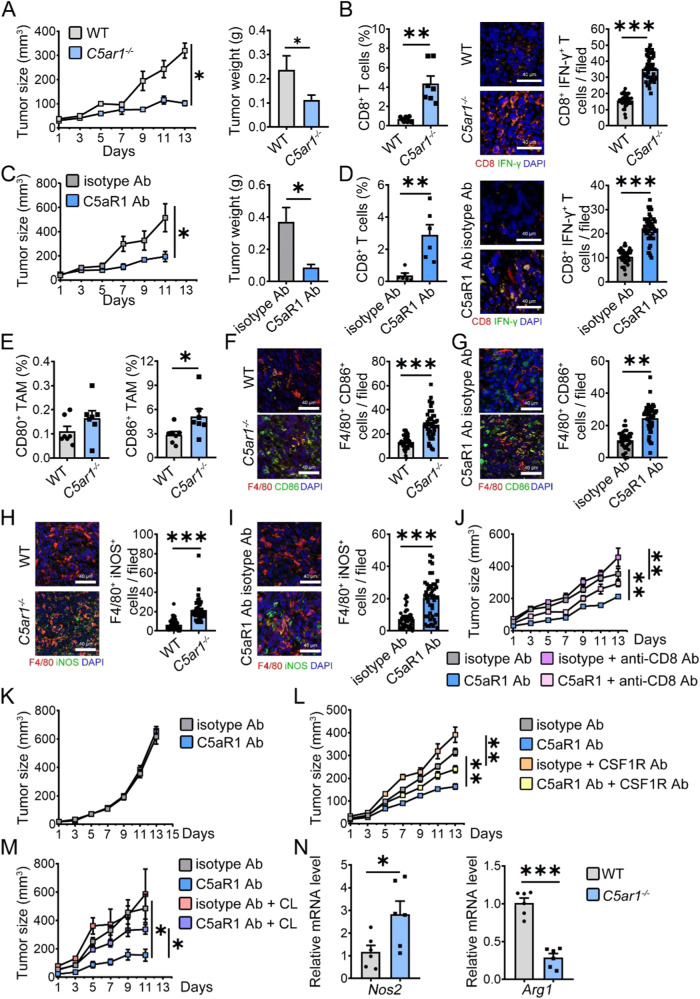
C5aR1 inhibition re-programs TAMs towards M1 phenotype. **A** Tumor growth was monitored in *C5ar1*^*+/+*^ mice (WT) and *C5ar1*^*-/-*^ mice bearing MC-38 cells. At the end of the experiment, the tumors were removed and weighed (*n* = 8). **B** The percentage of CD8^+^ T cells in CD45^+^ cells was analyzed by flow cytometry (*n* = 7, another tumor sample for pilot experiment of TAMs sorting) and the number of CD8^+^IFN-γ^+^ T cells was counted in tumor sections by Immunofluorescence staining (*n* = 3 tumor/group, 3 slide/tumor, 5 filed/slide). **C** Mice-bearing tumors were treated with either isotype control Ab or anti-C5aR1 Ab. At the end of the experiment, tumor growth and tumor weight were examined in the mice treated with either isotype control Ab (*n* = 8) or anti-C5aR1 Ab (*n* = 10). **D** The percentage of CD8^+^ T cells in CD45^+^ cells was analyzed by flow cytometry (*n* = 6, another two tumor samples for pilot experiment of TAM phagocytosis) and the number of CD8^+^IFN-γ^+^ T cells was counted by Immunofluorescence staining (n = 3 tumor/group, 3 slide/tumor, 5 filed/slide). **E** The percentages of CD80 and CD86 expressing on TAMs in tumors were analyzed by flow cytometry (*n* = 7). **F**–**I** The numbers of F4/80^+^CD86^+^ TAMs **F**, **G** and F4/80^+^iNOS^+^ TAMs (**H**, **I**) were counted in tumor sections from C5aR1 deficient mice or Ab-treated mice (*n* = 3 tumor/group, 3 slide/tumor, 5 filed/slide). **J** Mice-bearing tumors were treated with either isotype control Ab or anti-C5aR1 Ab in the presence of either isotype control Ab or anti-CD8 Ab. Tumor growth was assessed (anti-C5aR1 Ab group, *n* = 6, other groups, *n* = 8). **K** Tumor growth was examined in *Rag2*^*-/-*^ mice treated with or without anti-C5aR1 Ab (isotype control, *n* = 10, anti-C5aR1, *n* = 8). **L**, **M** Tumor growth was monitored in WT mice treated with or without anti-C5aR1 Ab in the presence of either anti-CSF1R Ab (**L**, *n* = 8/group) or clodronate liposomes (CL) (**M**, *n* = 6/group). **J**–**M** The data shown represent a single experiment. **N** Relative mRNA levels of the indicated genes were assessed by real-time RT-PCR (*n* = 6). **A**–**N** *, *P* < 0.05; **, *P* < 0.01; ***, *P* < 0.001.

C5aR1 plays a key role in regulating tumorigenesis in multiple ways including influencing the tumor microenvironment. While the underlying mechanisms of C5aR1 inhibition on TAM reprogramming needed to be further investigated. To further explore the effects of C5aR1 inhibition on reprogramming TAMs, we examined the infiltrated immune cells and found that compared with wild-type (WT, *C5ar1*^*+/+*^) mice and isotype antibody (Ab) treated mice, both *C5ar1* deletion and C5aR1 inhibition reduced tumor size and tumor weight along with an increase in the percentage of infiltrated CD8^+^ T cells and the number of CD8^+^IFN-γ^+^ cells (Fig. 1A–D). Meanwhile, the lack of C5aR1 increased the percentage of CD86 positive TAMs without influencing CD80^+^ TAMs (Fig. 1E). The number of F4/80^+^CD86^+^ cells was also elevated in tumors of either *C5ar1*^*-/-*^ mice or anti-C5aR1 Ab treated mice (Fig. 1F, G). Similarly, the number of TAMs expressing inducible nitric oxide synthase (iNOS) was also increased (Fig. 1H, I). The number of dendritic cells (DCs) did not change (Supplementary Fig. 1A), indicating that CD8^+^ T cells and TAMs were involved in C5aR1 inhibition-mediated tumor suppression.

To confirm the importance of CD8^+^ T cells and TAMs in C5aR1 inhibition-mediated tumor suppression, we first deleted CD8^+^ T cells and found that the lack of CD8^+^ T cells abolished the inhibitory effects of anti-C5aR1 Ab on tumor development (Fig. 1J, K, Supplementary Fig. 1B, C). Second, we deleted mono/macrophages and found that deletion of macrophages with anti-CSF1R Ab or clodronate liposomes (CL) ablated the inhibitory effect of anti-C5aR1 Ab on MC-38 colorectal cancer growth in parallel with a decrease in the infiltrated CD8^+^ T cells (Fig. 1L–M and Supplementary Fig. 1D–F). The mRNA levels of *Nos2* were increased, while the mRNA levels of *Arg1* decreased in TAMs (Fig. 1N), Furthermore, an OVA-mediated T-cell assay showed that deficiency or blockade of C5aR1 in macrophages promoted the proliferation of CD8^+^ T cells (Supplementary Fig. 1G). indicating that C5aR1 inhibition suppressed tumor growth by educating TAMs and enhancing TAM-mediated anti-tumor immunity of T cells.

Deletion and inhibition of C5aR1 increased the expression of CD86 on peritoneal macrophages belonging to the circulating mono/macrophages which are recruited locally and differentiate into TAMs [19] (Supplementary Fig. 1H). Moreover, phagocytosis of *C5ar1*^*-/-*^ BMDMs was elevated (Supplementary Fig. 1I). Our data demonstrated that C5aR1 deficiency or C5aR1 inhibition re-programmed TAMs to a tumor-inhibiting phenotype.

### Resetting M1 polarization by C5aR1 inhibition requires PFKM stabilization

**Fig. 2:**
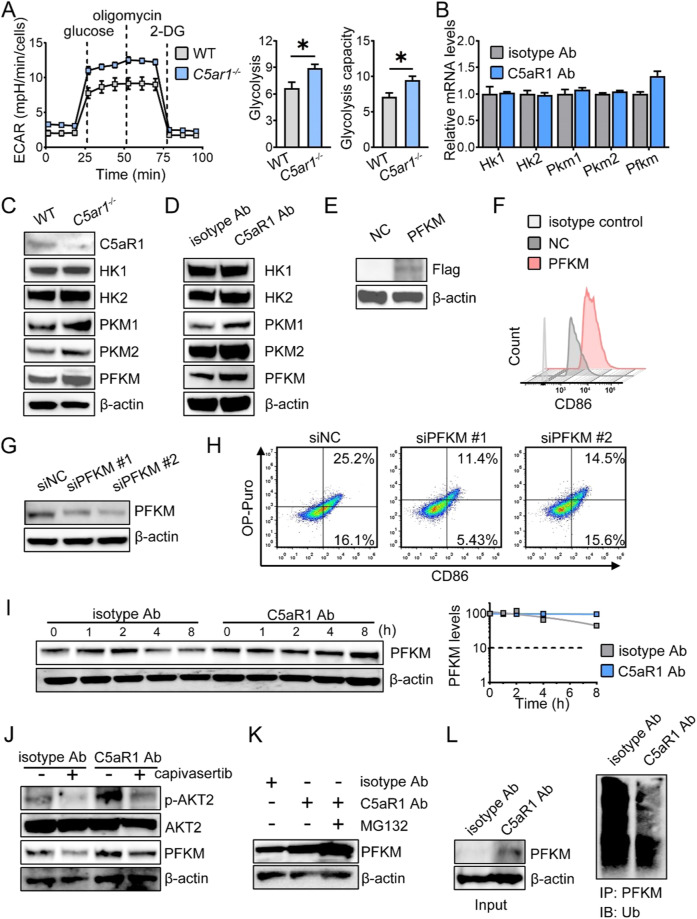
Resetting M1 polarization by C5aR1 inhibition requires PFKM stabilization. **A** The ECAR was determined by extracellular flux analysis, and the glycolysis and glycolysis capacity of the ECAR were quantified. The data shown represent a single experiment. **B** The relative levels of the indicated genes were quantified by real-time RT-PCR (*n* = 3). **C**–**E** The indicated protein expression levels were examined by Western blotting analysis. **F** The relative levels of CD86 were examined by flow cytometry (*n* = 2). **G** The indicated protein expression levels were examined by Western blotting analysis. **H** The relative levels of CD86 were examined by flow cytometry (*n* = 2). (**I**) BMDMs from CRC tumor mice were treated with CHX and harvested at the indicated time points. The relative levels of PFKM were quantified, normalized to actin, and plotted. **J** BMDMs from CRC tumor mice were treated with or without capivasertib (10 nM) for 18 h before harvesting. p-AKT2, AKT2, and PFKM were analyzed by Western blotting analysis. **K** BMDMs from Ab-treated CRC tumor mice were exposed to 10 μmol/L MG-132 for an additional 6 h before harvesting, and PFKM was analyzed by Western blotting analysis. **B**–**K** The figures shown are representative of one of two independent experiments. **L** The peritoneal macrophages from Ab-treated tumor mice were cocultured with MG-132. 6 h later, lysates were extracted, IP with an anti-PFKM Ab, and analyzed by Western blotting analysis using an anti-ubiquitin Ab. The data shown represent a single experiment. *P < 0.05.

Since the key enzymes involved in glycolysis are essential for M1 polarization [20]. We performed a Seahorse assay and examined the expression of key glycolytic enzymes. In comparison with WT BMDMs, both glycolysis and glycolytic capacity were increased in *C5ar1*^*-/-*^ BMDMs (Fig. 2A). In BMDMs, the ratio of OCR to EACR at the basal levels was decreased when C5aR1 was deleted or inhibited (Supplementary Fig. 2A, B). In *C5ar1*^*-/-*^ BMDMs, the mRNA levels of *Pfkm* were identical with that in WT BMDMs (Supplementary Fig. 2C). The mRNA levels of *Pfkm* were slightly elevated in BMDMs from anti-C5aR1 Ab-treated mice (Fig. 2B). C5aR1 deficiency or C5aR1 inhibition in vivo induced PFKM expression without affecting the expression of HK1/2 and PKM1/2 (Fig. 2C, D). We ectopically expressed PFKM in RAW264.7 cells (Fig. 2E). PFKM overexpression increased the level of CD86 (Fig. 2F), while PFKM silencing reduced CD86 expression (Fig. 2G, H), revealing that in vivo, C5aR1 deletion or C5aR1 inhibition triggered M1 polarization via inducing PFKM expression.

Since C5aR1 deletion or C5aR1 inhibition in vivo induced PFKM expression but barely affected *Pfkm* mRNA (Fig. 2B–D, Supplementary Fig. 2C), we assumed that C5aR1 inhibition-mediated PFKM expression via translation or post-translational modification. The mammalian target of rapamycin (mTOR) signaling pathway plays an important role in regulating protein synthesis. Therefore, we analyzed the downstream effectors of mTOR, and found that C5aR1 deletion did not influence mTOR pathway (Supplementary Fig. 2D), suggesting that PFKM expression could be induced by regulating stability rather than transcription or translation. As expected, blocking C5aR1 in vivo prolonged the half-life of PFKM (Fig. 2I).

AKT1 is involved in mediating the stabilization of phosphofructokinase 1 platelet isoform (PFKP) [21]. In contrast to AKT1, AKT2 activation is critical for M1 polarization and glycolysis [22]. We assumed that C5aR1 inhibition-mediated PFKM expression could be dependent on AKT2-regulated ubiquitination. Therefore, we first exposed BMDMs from either isotype control or anti-C5aR1 Ab-treated mice to capivasertib, a pan-AKT inhibitor. The level of p-AKT2 was increased, which was reduced when BMDMs were cocultured with capivasertib (Fig. 2J). Additionally, in BMDMs from anti-C5aR1 Ab-treated mice, PFKM expression was attenuated in the presence of capivasertib (Fig. 2J). We next challenged BMDMs from anti-C5aR1 Ab-treated mice with or without the proteasome inhibitor MG132. PFKM expression was further enhanced in the presence of MG132 in BMDMs from anti-C5aR1 Ab-treated mice (Fig. 2K). In addition, anti-C5aR1 Ab reduced PFKM ubiquitination in peritoneal macrophages from tumor-bearing mice (Fig. 2L), suggesting that upon C5aR1 inhibition or C5aR1 deletion, PFKM stabilization mediated by AKT2 activation was required for the M1 phenotype.

### C5aR1 inhibition resets macrophage polarization via activating TLR5/AKT2 pathway

**Fig. 3:**
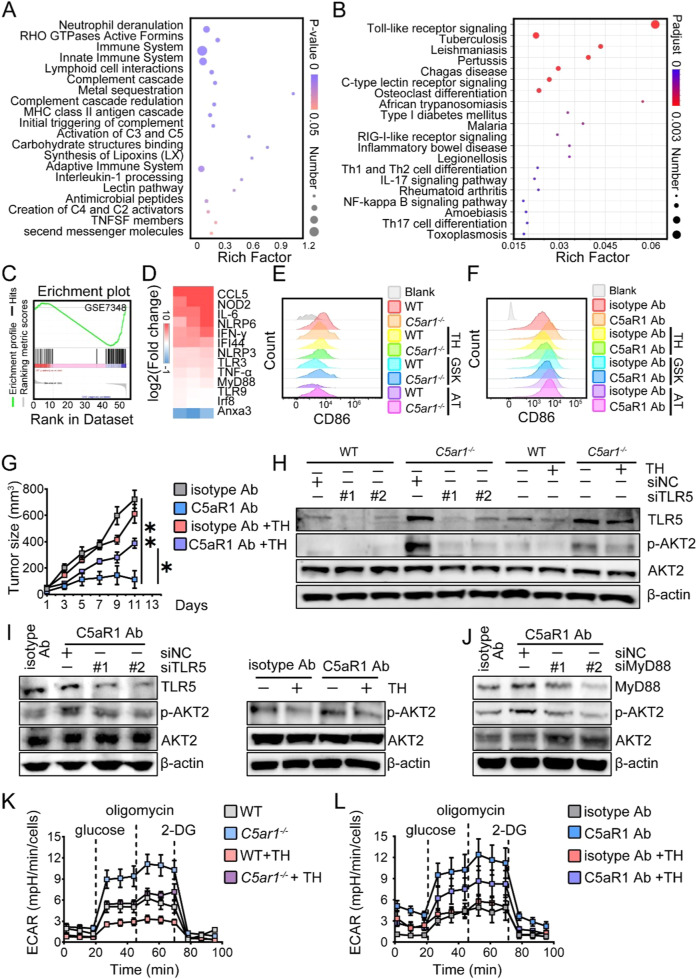
C5aR1 inhibition resets M1 macrophages via activating TLR5/AKT2 pathway. **A**, **B** GO analysis and KEGG analysis of gene sets involved in macrophage activation. **C** GSEA analyses of gene sets of macrophage activation. **D** Relative mRNA levels of the indicated genes in BMDMs from CRC tumor mice treated with either isotype control Ab or anti-C5aR1 Ab (*n* = 3). **E**, **F** Upon the indicated inhibitors, the relative expression of CD86 on BMDMs from C5aR1 deficient mice **E** or MC-38 syngeneic mice treated with the indicated Abs **F** was analyzed by flow cytometry (*n* = 2).TH, TH1020, GSK, GSK717, AT, AT791. **D**–**F** The results represent one of two or three independent experiments. **G** Tumor growth was assessed (*n* = 6/group). **H**–**J** Western blotting analysis. BMDMs of C5aR1 deficient mice **H** or MC-38 syngeneic mice treated with the indicated Abs (**I** and **J**) were cultured in the presence or absence of either siRNAs against the targets or TH1020 (1.5 μM). 24 h later, the levels of indicated proteins were analyzed by Western blotting analysis. The results represent one of two or three independent experiments. **K**, **L** The ECAR was determined by extracellular flux analysis. The data shown represent a single experiment. *P < 0.05.

To delineate the mechanisms underlying the re-programmed TAMs towards M1 phenotype in the absence of C5aR1 or C5aR1 inhibition, we determined the mRNA expression profile of BMDMs from WT and *C5ar1*^*-/-*^ mice. GO analysis showed that the gene profiles were quite different between these two groups, and the top 20 GO enrichments were mainly related to immune system processes (Fig. 3A). KEGG enrichment analysis revealed that the TLR-like receptor signaling pathway was the top 1 of the affected pathways (Fig. 3B). Gene set enrichment analysis (GSEA) showed that pathway of LPS-treated macrophages was upregulated in *C5ar1*^*-/-*^ BMDMs (Fig. 3C). The elevated gene expression in key immune regulatory networks was enriched in TLR signaling (e.g., *Ccl5*, *Cxcl5*), innate immunity, and polarization in BMDMs of MC38 syngeneic mice treated with anti-C5aR1 Ab (Fig. 3D).

To further investigate which TLRs are critical for M1 polarization in response to C5aR1 deletion or C5aR1 inhibition, we challenged BMDMs from either WT or *C5ar1*^*-/-*^ mice with TH1020 (TLR5 inhibitor), GSK717 (NOD2 inhibitor), or AT791 (TLR7/9 inhibitor). We found that TH1020, but not AT791 or GSK717 reduced CD86 expression (Fig. 3E). Similarly, when BMDMs from the indicated MC38 syngeneic mice were treated with TH1020, C5aR1 inhibition-induced CD86 expression were abolished (Fig. 3F). Moreover, the anti-tumor ability of anti-C5aR1 Ab was diminished in the presence of TH1020 in vivo (Fig. 3G and Supplementary Fig. 2E).

C5aR1 deletion or C5aR1 inhibition in vivo reprogrammed macrophages toward M1 polarization via AKT2 activation-mediated PFKM stabilization (Fig. 2). Therefore, we speculated that C5aR1 inhibition-mediated macrophage polarization depended on TLR5-mediated AKT2 activation. Silencing of TLR5 using siRNAs or pharmacological inhibition of TLR5 by TH1020 reduced p-AKT2 levels mediated by C5aR1 inhibition (Fig. 3H, I). Myeloid differentiation primary response 88 (Myd88) was involved in controlling p-AKT2 expression mediated by C5aR1 inhibition (Fig. 3J). The glycolysis in macrophages induced by C5aR1 suppression was decreased after TH1020 treatment (Fig. 3K–L and Supplementary Fig. 2F, G), indicating that C5aR1 inhibition promoted M1 polarization via activation of the TLR5/AKT2 signaling pathway.

### Gut microbiota plays a critical role in C5aR1 inhibition-triggered M1 polarization

**Fig. 4:**
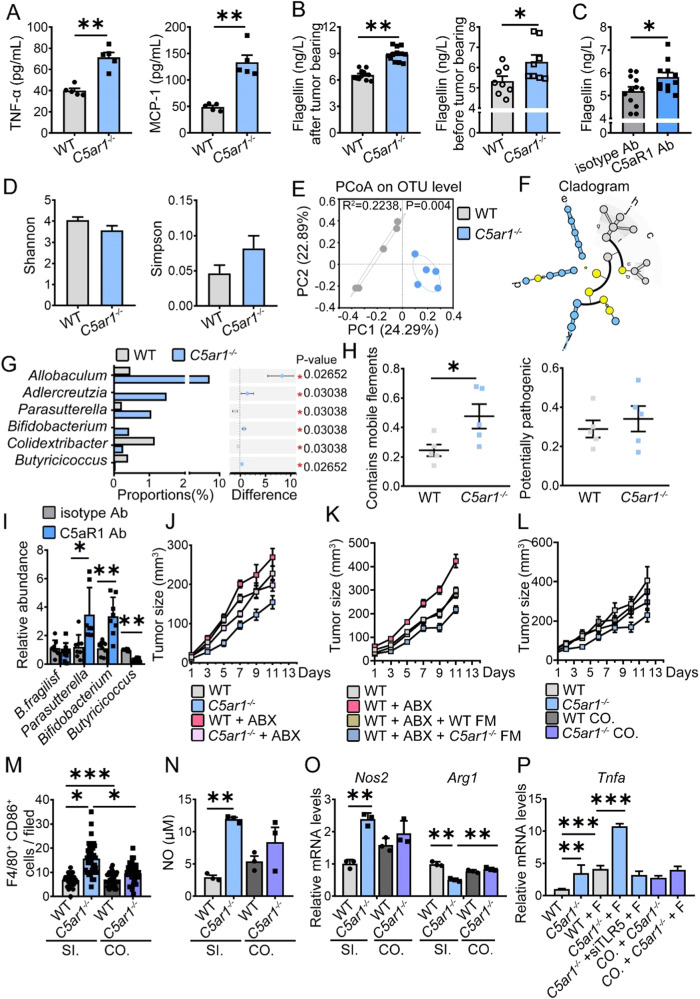
C5aR1 inhibition reset M1 polarization relies on modulating gut microbiota. **A** Serum samples were obtained from WT and *C5ar1*^*-/*-^ mice bearing MC-38 cells and subjected to examine the concentration of cytokines (*n* = 5/group). **B** Flagellin levels in the serum of WT and *C5ar1*^*-/*-^ mice with or without tumors were examined by ELISA (before tumor-bearing, *n* = 8; after tumor-bearing, *n* = 11 including 3 samples of a pilot experiment). **C** WT mice bearing MC-38 cells were injected with isotype control Ab or anti-C5aR1 Ab, and serum samples were obtained and subjected to ELISA to examine flagellin levels (*n* = 12 including 4 samples of the pilot experiment). **D** Shannon and Simpson diversities were assessed (*n* = 5). **E** principal coordinate analysis (PCoA) on the OUT level was examined (*n* = 5). Grey, WT; Blue, *C5ar1*^*-/-*^. **F** The differences between taxonomic or functional trees in two different groups (*n* = 5). **G** Wilcoxon rank-sum test bar plot on Genus level (*n* = 5). **H** The phenotype of mobile element initial for bacterial fitness and pathogenicity using the BugBase tool (*n* = 5). **D**–**H** The data shown are representative of one of two independent experiments. **I** The relative abundances of *Parasutterella*, *Bifidobacterium*, *B.fragilisf*, and *Butyricicoccus* were determined by real-time PCR (*n* = 8). The data shown represent a single experiment. **J** *C5ar1*^*+/+*^ and *C5ar1*^*-/-*^ mice were treated with ABX or normal water 3 weeks before tumor inoculation. The tumor growth and tumor weight were examined (*n* = 10). **K** MC-38 tumor growth in WT mice undergoing i.p. PMX-53 (1 mg/kg) with or without bacterial cocktail before tumor inoculation (ABX treated *C5ar1*^*-/-*^ mice, *n* = 6; others, *n* = 10/group). **L** *C5ar1*^*+/+*^ and *C5ar1*^*-/-*^ mice were housed alone or co-housed for 2 weeks prior to tumor inoculation. The tumor growth and tumor weight were examined (*n* = 14). **M** The numbers of F4/80^+^CD86^+^ TAMs were counted in tumor sections by Immunofluorescence staining (*n* = 3 tumor/group, 3 slide/tumor, 4 filed/slide). **M** The data shown represent a single experiment. **N** The concentrations of NO were assessed (*n* = 3). **O** Relative mRNA levels of the indicated genes were assessed by real-time RT-PCR (*n* = 3). **M**–**O** SI, single breeding, CO, co-cohoused breeding. **P** Relative mRNA levels of the *Tnfa* were assessed by real-time RT-PCR (*n* = 3). CO co-cohoused breeding, F flagellin. *P < 0.05; **P < 0.01; ***P < 0.001.

Since TLR5 is the receptor of bacterial flagellin. We first analyzed flagellin concentration. Besides cytokines such as TNF-α and MCP-1, the levels of serum flagellin were increased in *C5ar1*^*-/-*^ mice-bearing tumors (Fig. 4A, B). In addition, serum flagellin levels were elevated in *C5ar1*^*-/-*^ mice without tumors (Fig. 4B). Following the administration of anti-C5aR1 Ab, the serum levels of flagellin were also increased (Fig. 4C). Upon flagellin treatment, flagellin reduced tumor growth along with an increase in the concentration of TNF-α, and higher mRNA levels of *Tnfa* (Supplementary Fig. 3A–C), whereas the mRNA levels of *Arg1* were reduced (Supplementary Fig. 3C). Upon flagellin treatment, the levels of p-AKT2 were increased along with higher levels of PFKM (Supplementary Fig. 3D, E). Whereas, the Arg-1 levels were decreased (Supplementary Fig. 3D, E), indicating that upon C5aR1 inhibition, the release of flagellin molecules from dead bacteria could be involved in promoting M1 phenotype.

To confirm the role of bacterial flagellin in C5aR1 inhibition-regulated M1 polarization, we next examined the gut microbiota composition, and we found that compared with WT mice-bearing tumors, a difference in gut microbiota was observed in *C5ar1*^*-/-*^ mice-bearing tumors, although the Shannon and Simpson indices were barely affected (Fig. 4D, E). The relative abundance of the class Clostridia and the relative abundance of the family Lachnospiraceae was higher in WT mice bearing tumors (Fig. 4F and Supplementary Fig. 3F). In contrast, the abundance of the probiotic order Lactobacillales and family Lactobacillaceae was increased in *C5ar1*^*-/-*^ mice bearing MC-38 cells (Fig. 4F and Supplementary Fig. 3F). At the genus level, the relative proportions of *Allobaculum*, *Parasutterella*, and *Bifidobacterium* [23] which have anti-tumor properties, were significantly increased in *C5ar1*^*-/-*^ mice bearing tumors (Fig. 4G). Moreover, the initial phenotype of the mobile element for bacterial fitness was elevated without affecting pathogenicity (Fig. 4H). Similarly, upon anti-C5aR1 Ab treatment, the relative abundances of *Parasutterella* and *Bifidobacterium* were increased, and *Butyricicoccus* abundance was reduced (Fig. 4I).

To directly verify whether the gut microbiota was involved in regulating C5aR1 inhibition-modulated M1 polarization, we subsequently performed the antibiotic (ABX) cocktails experiment, fecal microbiota transplantation (FMT) experiment, and a cohousing experiment. We found that the reduced tumor growth by C5aR1 deficiency was abolished in the administration of the ABX cocktail (Fig. 4J and Supplementary Fig. 3G). Similarly, ABX treatment impaired the therapeutic effect of PMX-53 (Supplementary Fig. 3H). FMT experiment demonstrated that prophylactic transfer of *C5ar1*^*−/−*^ microbiota was able to suppress tumor growth (Fig. 4K and Supplementary Fig. 3I). In addition, CRC growth was no longer inhibited in *C5ar1*^*-/-*^ mice that co-housed with WT mice (Fig. 4L). The number of CD86^+^ TAMs and the NO concentration in TAMs were elevated in *C5ar1*^*-/-*^ mice bearing tumors, which were decreased when *C5ar1*^*-/-*^ mice were co-housed with WT mice (Fig. 4M, N). The mRNA levels of *Nos2* were increased, while the mRNA levels of *Arg1* decreased in TAMs isolated from *C5ar1*^*-/-*^ mice bearing tumors, which was reversed when *C5ar1*^*-/-*^ mice were co-housed with WT mice (Fig. 4O). In vitro experiments also revealed that the mRNA level of *Tnfa* was rescued when BMDMs of *C5ar1*^*-/-*^ co-housed mice were treated with flagellin (Fig. 4P). Collectively, these observations suggested that C5aR1 inhibition-mediated macrophage M1 polarization depended on the gut microbiota.

### Resetting M1 polarization by C5aR1 inhibition relies on IL-22-modulated gut microbiota

**Fig. 5:**
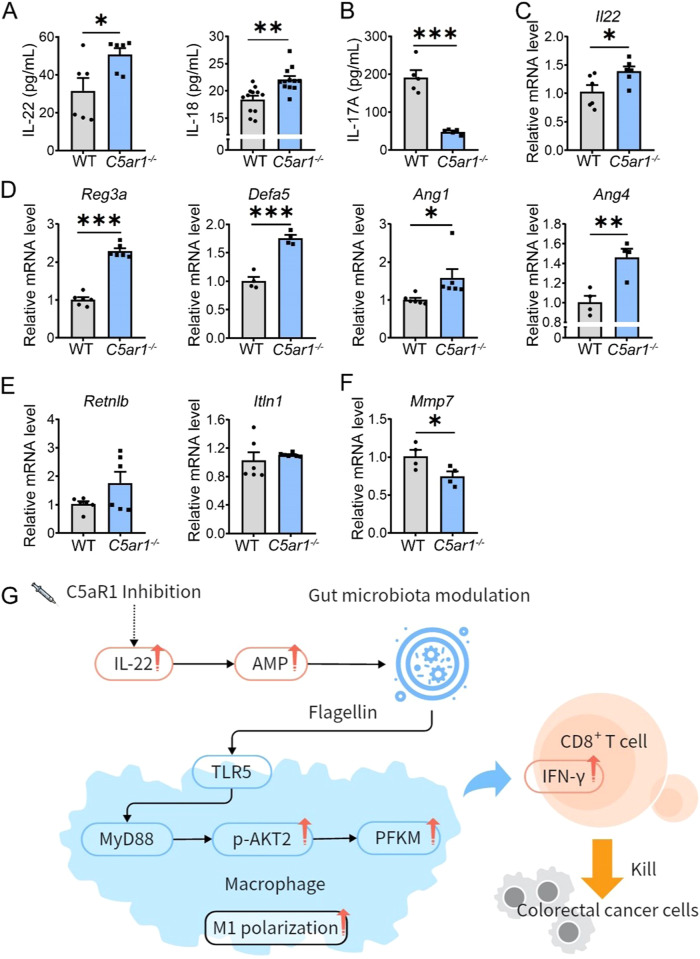
C5aR1 inhibition reset M1 macrophages could rely on IL-22-modulated gut microbiota. **A**, **B** Serum samples were obtained and subjected to ELISA to examine IL-22 (*n* = 6), IL18 (*n* = 12), and IL-17 levels (**B**, *n* = 5). **C** Relative mRNA levels of the *Il22* were assessed by real-time RT-PCR (*n* = 6). (**D**–**F**) Relative mRNA levels of the indicated genes were assessed by real-time RT-PCR (*n* = 4 or *n* = 6). **G** Upon C5aR1 inhibition, IL-22-induced AMPs promoted flagellin release from dead bacteria. Serum flagellin was increased along with the activation of TLR5/AKT2 signaling, leading to PFKM stabilization and the M1 phenotype, which in turn suppressed CRC growth.

It has been reported that cytokines such as IL-22 and IL-17 are required for shaping gut flora via inducing AMPs and promoting epithelial barrier function [24–27]. IL-22 harbors more potent in AMPs induction [28]. We therefore assessed the cytokines production and found that the levels of IL-22 and IL-18, but not IL-17 were increased in *C5ar1*^*-/-*^ mice (Fig. 5A, B). Group 3 innate lymphoid cells (ILC3) and Th17 cells are the main producers of IL-22 [29]. Real-time RT-PCR showed that *Il22* mRNA levels were elevated (Fig. 5C and Supplementary Fig. 4). Moreover, the mRNA levels of AMPs encoded genes including regenerating family member 3α (*Reg3a*) and defensin α5 (*Defa5*) were increased in *C5ar1*^*-/-*^ IECs (Fig. 5D). The mRNA levels of Angiogenin-1 (*Ang1*) and *Ang4* that can be induced by IL-22 [30] were also increased in *C5ar1*^*-/-*^ IECs (Fig. 5D). The mRNA levels of *Retn1b* and antimicrobial protein intelectin 1 (*Itln1*) induced by IL-18 [31] were almost identical in *C5ar1*^*-/-*^ IECs (Fig. 5E). Meanwhile, the mRNA levels of *Mmp7* that is the downstream target of IL-17 [32, 33] were reduced in *C5ar1*^*-/-*^ IECs (Fig. 5F). These observations indicated that IL-22-regulated AMPs could drive the release of flagellin from dead bacteria, which contributed to C5aR1 inhibition-mediated M1 polarization.

### An increase of C5aR1 in TAMs predicts a poor prognosis

To assess the role of C5aR1 in CRC development, we examined the relative expression of C5aR1 in TAMs of CRC patients using single-cell RNA-seq data visualization and analysis (SCDVA, http://crcleukocyte.cancer-pku.cn/) [34]. We found that C5aR1 was highly expressed in myeloid cells, especially in TAMs (Supplementary Fig. 5A). We also assessed C5aR1 expression in immune cells and found that in normal human and mouse tissues (http://biogps.org), C5aR1 was aberrantly expressed in macrophages (Supplementary Fig. 5B). We confirmed that in comparison with para-carcinoma samples, C5aR1 was abundant in both tumor cells and TAMs (Supplementary Fig. 5C). Notably, it seemed that CRC patients with low C5aR1 expression in TAMs exhibited longer overall survival when compared to CRC patients with high C5aR1 expression in TAMs (Supplementary Fig. 5C). These data suggest that the aberrant accumulation of C5aR1 in TAMs may be critical for tumor growth.

Taken together, our data indicated that the expression of C5aR1 in TAMs could predict the prognosis of CRC patients. In addition, upon C5aR1 deletion or C5aR1 inhibition in vivo, IL-22-induced AMPs led to the release of flagellin from dead bacteria, activating TLR5/AKT2 signaling pathway, which promoted PFKM stabilization and the M1 phenotype, and subsequently enhanced T cell-mediated anti-tumor immunity and suppressed CRC growth (Fig. 5G).

## Discussion

C5a/C5aR1 signaling has been shown to promote tumor progression by recruiting MDSCs in breast malignancies [35]. In addition, C5a/C5aR1 drives the production of immunosuppressive cytokines and creates a pre-metastatic niche microenvironment [36]. Switching to M1 macrophages may be a promising treatment strategy for cancer immunotherapy. Macrophage polarization and function are affected by metabolism and metabolites. The blockade of C5aR1 decreases oxidative phosphorylation (OXPHOS) [37]. In the present study, we found that C5aR1 inhibition increased the expression of PFKM the key enzyme critical for glycolysis via TLR5-mediated AKT2 activation in TAMs; thus, rendering TAMs susceptible to M1 polarization (Figs. 1–3).

In our present study, we found that the absence of C5aR1 caused PFKM upregulation and glycolytic metabolism change in the original state of BMDMs (Fig. 2). A growing number of studies have raised the concept of “trained immunity” in which macrophages can acquire memory-like characteristics with metabolic and epigenetic reprogramming, and the immunological phenotype of trained macrophages has been proven to last at least 3 months and up to 1 year [38, 39]. Therefore, the original state of BMDMs can be different when they derived from mice that received different treatments.

Bacterial flagellin, the principal component of the flagellum, can be recognized by several cells including macrophages and neutrophils, via the transmembrane receptor TLR5. In our study, we found that inhibition of C5aR1 and deletion of C5aR1 increased serum flagellin concentration and induction of the M1 phenotype (Figs. 1–4 and Supplementary Fig. 3). Treatment of BMDMs with flagellin induced activation of the TLR5 signaling pathway, and M1 marker expression (Supplementary Fig. 3D, E). In contrast, blockade of TLR5 attenuated C5aR1 inhibition-regulated tumor suppression in association with a decrease in CD86 expression and glycolysis in macrophages (Figs. 3E–G, K, L). These data revealed that C5aR1 inhibition*-*induced flagellin release from dead bacteria could be the accelerator that drives the M1 phenotype and enhances T-cell-mediated anti-tumor immunity.

The abundance of *Bifidobacterium* is positively related to CD8^+^ T-cell infiltration [40], and *Bifidobacterium* inhibits tumor growth by enhancing T-cell anti-tumor immunity [41]. In this study, we also found that at the genus level, the relative proportions of probiotics, including *Allobaculum*, *Parasutterella*, and *Bifidobacterium* were increased in *C5ar1*^*-/-*^ mice bearing tumors (Fig. 4G). Consistently, C5aR1 inhibition in vivo also increased the relative abundances of *Parasutterella* and *Bifidobacterium*. These data suggest that C5aR1 deficiency and C5aR1 inhibition in vivo modulated the gut microbiota composition.

Gut flora can be shaped by several cytokines special IL-22 via controlling AMP production [28]. Our data showed that the serum concentration of IL-22 was elevated in parallel with an increase in the mRNA levels of AMPs (Fig. 5A, C), which could lead to an increase in flagellin release from dead bacteria. It has been shown that C5a is tented to enhance TLR2 signaling [42] which was involved in IL-22 generation [43, 44]. We found that in ILC3 cells isolated from C5aR1 KO mice, upon C5a treatment, the levels of *Il22* mRNA were obviously increased, which were blocked by Cucpt22 (Supplementary Fig. 4). The role of IL-22 in cancer immunity is controversial. It has been shown that IL-22 induced proliferation of human CRC cells via STAT3-dependent signaling [45]. While, in an AOM/DSS-induced CRC model, deletion of IL-22 increased in tumor number and tumor size [46]. The diverse roles of IL-22 in shaping gut flora in response to C5aR1 inhibition need to be further investigated.

Several inhibitors targeting C5aR1, such as MOR210, have been developed and are currently in phase I clinical trials for solid tumors (NCT04678921). Our results provide evidence that C5aR1 is aberrantly expressed in TAMs; inhibition of C5aR1 promotes M1 polarization via TLR5-mediated PFKM expression. Given that human CRC was highly infiltrated with C5aR1^+^ TAMs, and that an increase in survival was observed in CRC patients with C5aR1^-^ TAM tumors, we asserted that these malignancies could benefit from therapies targeting C5aR1.

## Materials and methods

### Mice

C57BL/6 (B6) (CD45.2), *Rag2*^*-/-*^, and *C5ar1*^*-/-*^ female mice at 6-8 weeks old were obtained from the Jackson Laboratory (Ellsworth, Maine, US). Mice were maintained and housed under specific pathogen-free (SPF) conditions. And mice were grouped randomly. At the end of the experiment, the mice were terminated humanely. The investigator was not blinded to the group allocation of the animals during the experiment. No statistical method was used to predetermine the sample size for the mice experiment, which was based on preliminary experimental results. The sample size of each experiment is shown in the legend. No data were excluded from the analysis. The mice were strictly bred and maintained according to protocols approved by the Institutional Animal Care and Use Committee of Xuzhou Medical University (Approval No. 202111A020 and 202202A021).

### Ectopic tumor implantation and weight measurements

Tumor implantation and weight measurements were performed as previously described [47].

For anti-C5aR1 Ab treatment, mice were treated with either the same volume of isotype control Ab or 1 mg/kg anti-C5aR1 mAb (clone 20/70, Cedarlane, Ontario, Canada) by intravenous (i.v.) injection on Day −1 and Day 7.

For PMX-53 treatment, mice were treated with either PBS or 1 mg/kg PMX-53 by i.v. every other day.

For flagellin treatment, mice were divided into two groups and treated with PBS or 10 μg/mouse flagellin (PrimeGene, Shanghai, China) by intraperitoneal injection on Day −1 and Day 7.

CD8^+^ T cell deletion was executed as described [47].

Macrophage depletion by anti-CSF-1R Ab (Leinco Technologies, Missouri, USA) or clodronate-loaded liposomes (Liposoma, Netherlands) was performed as described [48].

The in-house experiment and FMT experiment were performed as described [49]. For the FMT experiment, in brief, 100 mg feces from *C5ar1*^*-/-*^ or WT mice were given to mice treated with ABX. 2 weeks later, mice were implanted with MC-38 cells and given the fecal microbiome from *C5ar1*^*-/-*^ or WT mice for another 2 weeks.

The antibiotics treatment experiment was explored as described [50]. Briefly, 0.25 g Vancomycin (Macklin, Shanghai, China), 1 g penicillin (Macklin), 1 g neomycin (VICMED, Xuzhou, China), and 1 g metronidazole (VICMED) were dissolved in 1 liter (L) sterile drinking water. Solutions and bottles were changed 2–3 times per week.

### Cell isolation and culture

Bone marrow-derived macrophages (BMDMs), 293 T, and RAW264.7 cells were cultured as described [51]. Murine intestinal cells were isolated as described [49]. TAMs were harvested as described [52]. To obtain type 3 innate lymphoid cells (ILC3s), the intestine from mice was rinsed with PBS and then digested with RPMI-1640 containing 1 mg/mL collagenase IV (VICMED), 20 ng/mL DNase I (VICMED) at 37 °C for 45 min. The tubes were gently vortexed every 8–10 min. The cells were washed with fresh PBS, strained through a 70 µm cell strainer, and then stained with antibodies of ILC3 makers. Lin^-^ CD45^+^ CD127^+^ CD25^+^ ST2^-^ CCR6^+^ cells were sorted by flow cytometry, and cultured with RPMI-1640 containing 10% FBS.

### Reagents

PMX-53, TH1020 (TLR5 inhibitor), AT791 (TLR7/9 inhibitor), GSK717 (NOD2 inhibitor), cycloheximide (CHX), oligomycin, and MG-132 were obtained from MCE (Shanghai, China). 2-deoxy-D-glucose (2-DG) and glucose were purchased from VICMED.

### siRNAs, plasmid DNA, and transfection

siRNAs against PFKM (#1, 5′-GCUGAAUGAUCUCCAGAAATT-3′, 5′-UUUCUGGAGAUCAUUCAGCTT-3′; #2, 5′-GGACCAGACAGACUUUGAATT-3′, 5′-UUCAAAGUCUGUCUGGUCCTT-3′); TLR5 (#1, 5′-CGGGAACUGAAUUCCUUAATT-3′; 5′-UUAAGGAAUUCAGUUCCCGTT-3′; #2, 5′-GACCAAACAUUCAGAUUAUTT-3′; 5′-AUAAUCUGAAUGUUUGGUCTT-3′) and siMyd88 (#1, 5′-GCCUAUCGCUGUUCUUGAATT-3′; 5′-UUCAAGAACAGCGAUAGGCTT-3′; #2, 5′-CCAACGAUAUCGAGUUUGUTT-3′; 5′-ACAAACUCGAUAUCGUUGGTT-3′) was purchased from Jima (Shanghai, China). BMDMs were transfected with the indicated siRNAs as described [51]. After 24 h, cells were harvested and subjected to the indicated experiments.

### Plasmid DNA, lentivirus, and infection

Murine C5aR1 lentivirus was purchased from Obiosh (Shanghai, China). The murine PFKM cDNA was amplified and cloned into the pLVX-IRES-Zsgreen1 vector. Lentiviruses were generated and stable cell lines were established as described previously [53].

### Surface staining and flow cytometry analysis

The BM samples were stained with the indicated antibodies and analyzed using a FACSAria flow cytometer (BD Biosciences) [54].

For peritoneal lavage analysis, 5 mL of PBS was injected into the peritoneal cavity and the abdomen was gently massaged. After washing, nonspecific antibody binding to the cells was blocked and stained with the indicated antibodies.

The infiltrated immune cells in the tumor tissues were prepared as previously described [55]. The cells were stained with the indicated antibodies, and 7-AAD was used to label dead cells. The antibodies used are listed in Supplementary Table S1. All data were analyzed using FlowJo software (FlowJo LLC).

### Multiplexed immunofluorescence staining and confocal microscopy

A formalin-fixed, paraffin-embedded CRC tissue microarray was purchased from Shanghai Outdo and stained with the indicated antibodies using a PANO 7-plex IHC kit (PANO, Beijing, China). Images were acquired and analyzed using TissueFAXs and StrataQuest tissue analysis software (TissueGnostics, Beijing, China) as described [56].

Sections (5 μm) of paraffin-embedded tissues were stained with the indicated antibodies using a PANO 7-plex IHC kit (PANO), as previously described [56]. The analyses were performed using confocal laser scanning microscope (CLSM, Leica STELLARIS 5, Germany). The antibodies used are listed in Table S1.

### T cell proliferation assay

Naive CD8^+^ T cells were sorted from the spleens and lymph nodes of OT-I mice by FACS, labeled with 2.5 µM CFSE for 10 min, and cocultured with BMDMs pulsed with 3 µg/mL SIINFEKL peptide (OVA#SLBQ9036V). Three days later, the proliferation of CD8^+^ OT-I cells was analyzed by flow cytometry.

### Phagocytosis assay

MC-38 cells were labeled with 2.5 µM CFSE (BD Biosciences) for 10 min and co-cultured with TAMs. After 48 h, cells were collected and subjected to flow cytometry to analyze the phagocytosis of TAMs.

### Metabolic assays

The extracellular acidification rate (ECAR) and oxidative phosphorylation (OCR) were measured using an XF 24 extracellular flux analyzer (Seahorse Bioscience) as described [53]. The measurements were normalized to the cell number.

### Newly synthesized CD86 protein examination

BMDMs were transfected with siNC or the indicated siRNA. After 24 h, the cells were treated and subjected to flow cytometry to analyze the newly synthesized CD86 according to the manufacturer’s protocol (ab235634, Cambridge, CB2 0AX, UK).

### C5aR1 expression

*C5aR1* mRNA expression in different mouse cells/tissues or different human cells, as per BioGPS (http://biogps.org). The online database Gene Expression Profiling Interactive Analysis (GEPIA2, http://gepia2.cancer-pku.cn/#index) was used to analyze the survival analysis for C5aR1 expression with CRC.

### Microarray analysis

Total RNA was isolated from the BMDMs of *C5aR1*^*+/+*^ and *C5aR1*^*-/-*^ mice using TRIzol reagent (Invitrogen) according to the manufacturer’s protocol. Library preparation, clustering, and sequencing were performed by MajorBio (Shanghai, China).

### Real-time RT-PCR

The total RNA of TAMs was extracted using the Cell-to-CT 1-step Power SYBR Green kit (Invitrogen) as described [52]. Total RNA was isolated, and cDNAs were synthesized and subjected to real-time RT–PCR as described [56]. The primers used for PCR are listed in Table S2.

### Fecal DNA extraction and quantification

Fecal DNA was extracted using the CWBIO Stool Genomic DNA Kit (CWBIO, China) according to the manufacturer’s protocol, and the concentration was measured by Nanodrop Lite (Thermo). The quantitative PCR assays were performed using the LightCycler 480 SYBR Green Master Mix (Roche (USA) 04887352001). The primers used for PCR are listed in Table S3.

### Fecal 16 S rRNA microbial analysis

Fresh feces collected from individual mice were stored at −80 °C until analysis. Fecal DNA extraction and sequencing were performed by Majorbio (Shanghai, China) as described [57]. Data processing was performed as described [57].

### Immunoblot assay

Immunoblotting was performed as previously described [58]. The antibodies used are listed in Table S1.

### In vivo Ubiquitination Assay

Proteins of sorted macrophages from peritoneal lavage were extracted and subjected to immunoprecipitation as described [58]. Western blotting was performed to probe ubiquitinated forms of PFKM.

### Protein half-life assay

Protein half-life assay was performed as described [58].

### Nitric oxide (NO) concentration and cytometric bead array (CBA)

The concentrations of NO and cytokines were assessed using a Nitric Oxide Assay Kit (Beyotime Biotechnology, Shanghai, China) or a CBA kit (BioLegend), according to the manufacturer’s instructions.

### Enzyme-linked immunosorbent assay (ELISA)

The concentration of MCP-5 and flagellin in the serum was examined using ELISA kits (Jiangsu Meimian Industrial Co., Ltd., Yancheng, China) according to the manufacturer’s instructions.

### Statistical analysis

Experiments using flagellin, anti-CSF1R, or anti-CD8 antibodies, ABX treatment, and FMT were performed once. The other mouse experiments were repeated twice and the data were combined. The data were processed using SPSS25.0 statistical software, and the measurement data are expressed as mean ± standard deviation (x ± s). Two groups with normality and homogeneity of variance were tested using independent samples. One-way ANOVA followed by LSD was used to test pairwise comparisons between groups. Based on the median number of C5aR1^+^ TAMs in CRC tissues. Seventy-seven CRC samples were divided into a high C5aR1 expression group (*n* = 38) and a low C5aR1 expression group (*n* = 39). The relationship between C5aR1 expression levels in TAMs and the survival rate of patients was analyzed using the log-rank (Mantel-Cox) test. The hypothesis testing level was set at α = 0.05, and *P* < 0.05. Statistical graphs were plotted using the GraphPad Prism software (version 9.0).

### Supplementary information

supplemently material

Original Data File

checklist

## Data Availability

All data generated during this study are included either in this article or in the supplementary information files. The gene expression data has been deposited in the GEO database under the accession number GSE252048.
