# Supplementary material for: Blockade of C5aR1 resets M1 via gut microbiota-mediated PFKM stabilization in a TLR5-dependent manner
Source: Cell Death Dis. 2024 Feb 8;15(2):120. doi: 10.1038/s41419-024-06500-4 (PMC10853248; doi:10.1038/s41419-024-06500-4)
Supplement: Supplementary file 1 — supplemently material [file 41419_2024_6500_MOESM1_ESM.docx]

**Supplementary Fig.1**


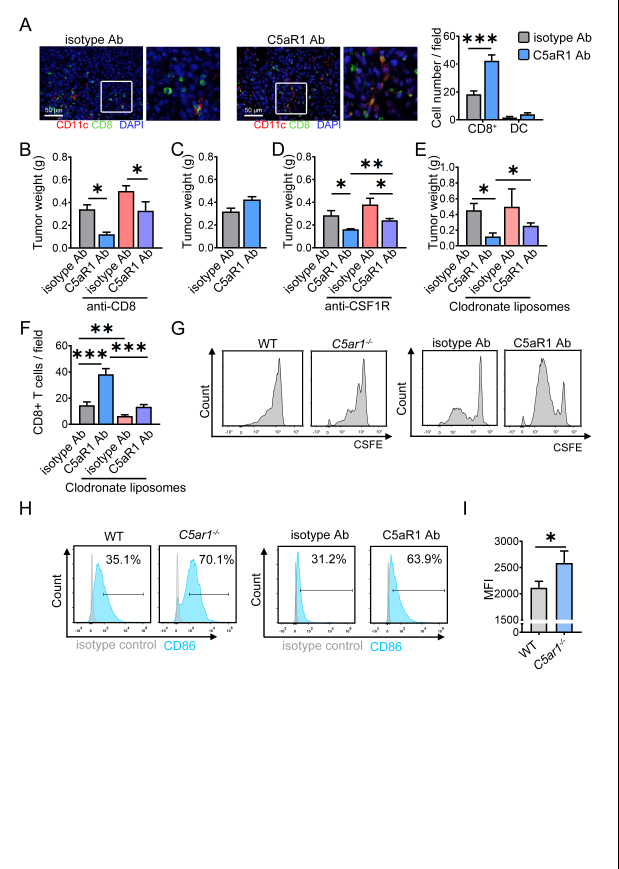


**Supplementary Fig.2**


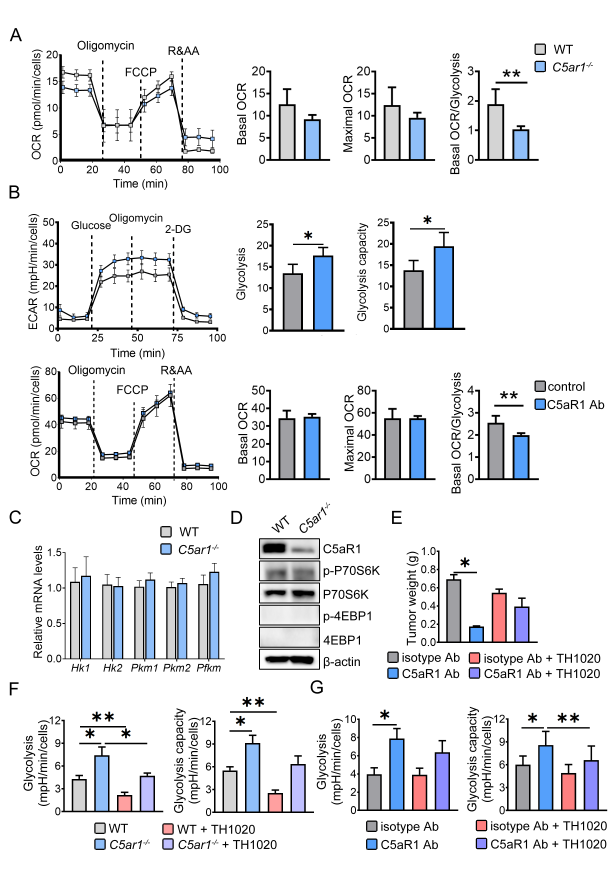


**Supplementary Fig.3**


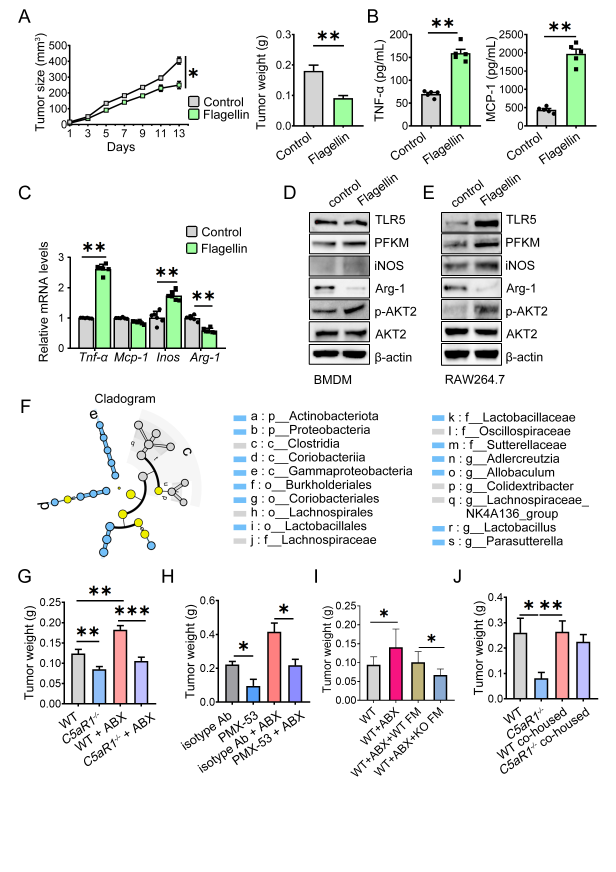


**Supplementary Fig.4**


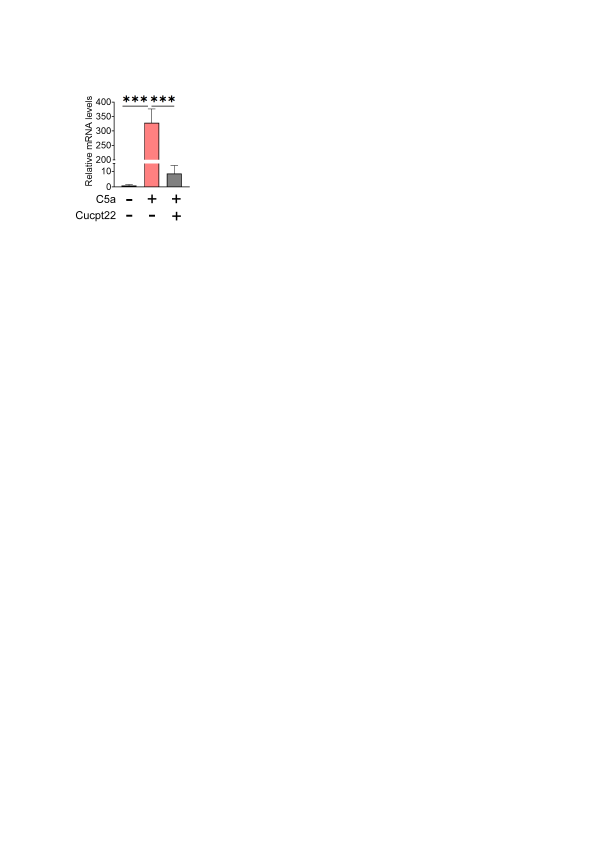


**Supplementary Fig.5**


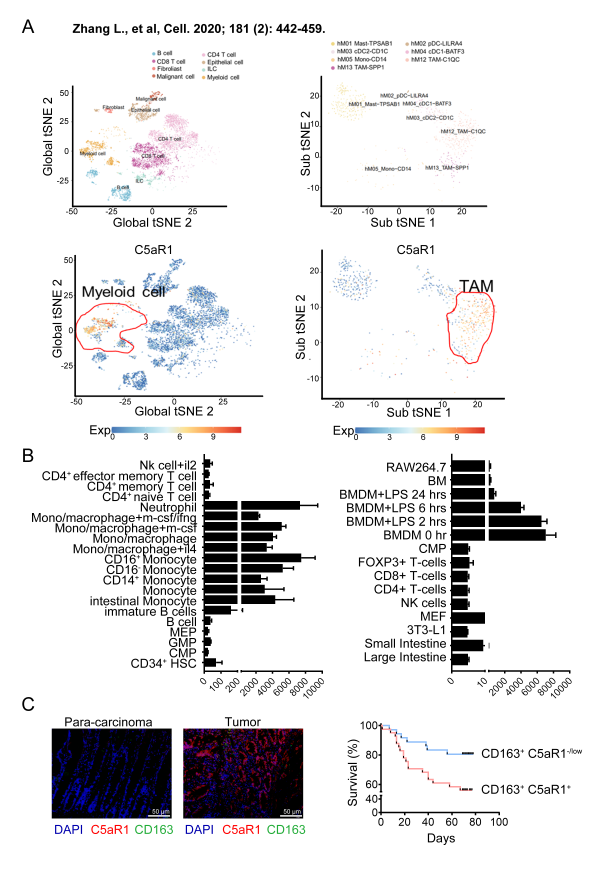


**Supplementary Fig.1 C5aR1 inhibition re-programs TAMs towards M1 phenotype and promotes T cell proliferation.**

(A) Immunofluorescence for CD8 (green), CD11c (red), and DAPI (blue) in tumor sections was performed. (B-E) The tumor weights were examined. (F) The number of CD8^+^ T cells was counted in tumor sections by Immunofluorescence staining. (G) The OVA-mediated T-cell proliferation was analyzed by flow cytometry. (H) The percentages of CD86^+^ macrophages in peritoneal lavage were analyzed by flow cytometry. (I) The phagocytosis of macrophages was assessed by flow cytometry.

**Supplementary Fig.2 C5aR1 inhibition re-programs TAMs towards M1 phenotype via activation of the TLR5/AKT2 signaling pathway.**

(A) BMDMs were treated by sequential injection of following compounds: oligomycin (2 μmol/L), carbonyl cyanide-4 (trifluoromethoxy) phenylhydrazone (FCCP, 4 μmol/L) and antimycin A (1 μmol/L) plus rotenone (100 nmol/L), the basal respiration and maximal respiration of the OCR were quantified. The ratio of basal respiration and glycolysis was analyzed. The data shown represent a single experiment. (B) The ECAR and OCR were determined by extracellular flux analysis, and the glycolysis and glycolysis capacity of the ECAR were quantified. The basal respiration and maximal respiration of the OCR were quantified. And the ratio of basal respiration and glycolysis was analyzed. The data shown represent a single experiment. (C) The relative levels of the indicated genes were quantified by real-time RT-PCR (n = 3). (D) The indicated protein expression levels were examined by Western blotting analysis (E) The tumor weights were examined. (F-G) The glycolysis and glycolysis capacity were analyzed.

**Supplementary Fig.3 Bacterial flagellin is required for C5aR1 inhibition-reset M1 phenotype.**

(A) WT mice bearing MC-38 cells were i.p. injected with PBS or flagellin (10 μg/mouse). The tumor growth was monitored, and tumor weights were examined at the end of the experiment (ctrl, n = 8; flagellin, n = 6). (B) Cell culture supernatant samples were obtained from BMDMs treated with or without flagellin (100 ng/mL) and subjected to analyze the concentration of cytokines (n = 5). (C) Relative mRNA levels of the indicated genes were assessed by real-time RT-PCR (n = 6). (D-E) The indicated protein expression levels were examined by Western blotting analysis. (F) The differences between taxonomic or functional trees in two different groups (n = 5). (G-J) The tumor weights were examined. In H, MC-38 tumor growth in WT mice undergoing i.p. PMX-53 (1mg/kg) with or without bacterial cocktail before tumor inoculation (n = 10). *, *P* < 0.05, **, *P*< 0.01.

**Supplementary Fig.4 C5aR1 inhibition reset M1 macrophages could rely on ILC3s derived IL-22.**

The *C5ar1^-/-^* ILC3 were sorted by FACS, and subjected to C5a treatment (10 ng/mL) or the same volume of control diluent in the absence or presence of Cucpt22 (5 μM) for 24 h. The relative mRNA levels of *Il22* were quantified by real-time RT-PCR (n = 3).

**Supplementary Fig.5 Increase of C5aR1 in TAMs predicts a poor prognosis.**

(A) The relative expression of C5aR1 on TAMs was analyzed. (B) The distribution of C5aR1 in human and mouse tissue/cells. (C) Immunofluorescence for C5aR1 (red), CD163 (green), and DAPI (blue) in tumor sections were performed (n=77). Kaplan–Meier curves for progression-free survival probability in CRC patients.

**Table S1.** Antibody Information.

| Antibody | Cat number | Company | Dilution factor |
| --- | --- | --- | --- |
| anti-PE-Cy7-CD16/32 | Cat: 101318 | BioLegend | 1:200 |
| anti-BV510-F4/80 | Cat: 123135 | BioLegend | 1:200 |
| anti-FITC-CD11b | Cat: 101206 | BioLegend | 1:200 |
| anti-Percp-Cy5.5-CD45 | Cat: 103132 | BioLegend | 1:200 |
| anti-APC-CD127 | Cat: 121122 | BioLegend | 1:200 |
| anti-PE-IL-33Ra | Cat: 146607 | BioLegend | 1:200 |
| anti-PE/Cy7-CD25 | Cat: 552880 | BioLegend | 1:200 |
| anti-BV421-CD196 | Cat: 129817 | BioLegend | 1:200 |
| anti-FITC-CD80 | Cat: E-AB-F0992UC | Elabscience | 1:200 |
| anti-APC-CD86 | Cat: E-AB-F0994UD | Elabscience | 1:200 |
| anti-APC-Cy7-CD45 | Cat: 561037 | BD Biosciences | 1:200 |
| anti-PE-Cy7-CD11c | Cat: 558079 | BD Biosciences | 1:200 |
| anti-BV510-F4/80 | Cat: 553036 | BioLegend | 1:200 |
| anti-PerCP-CD8 | Cat: 553036 | BioLegend | 1:200 |
| anti-PE/Cy7-TCRβ | Cat: 109222 | BioLegend | 1:200 |
| anti-PE-MHCⅡ | Cat: EAB-F0990UD | Elabscience | 1:200 |
| anti-CD163 | Cat: 16646-1-AP | Protaintech | 1:100 |
| anti-CD68 | Cat: 25747-1-AP | Protaintech | 1:100 |
| anti-C5aR1 | Cat: 21316-1-AP | Protaintech | 1:2000 |
| anti-F4/80 | Cat: D2S9R | CST | 1:100 |
| anti-IFN-γ | Cat: 9134 | Abcam | 1:100 |
| anti-CD86 | Cat: E5W6H | CST | 1:100 |
| anti-CD8 | Cat: D4W2Z | CST | 1:100 |
| anti-INOS | Cat: 80517-1-RR | Protaintech | 1:2000 (WB)  1:100 (IF) |
| anti-Arg1 | Cat: SY09-06 | HUABIO | 1:2000 |
| anti-HK1 | Cat: 19662-1-AP | Proteintech | 1:2000 |
| anti-HK2 | Cat: 22029-1-AP | Proteintech | 1:2000 |
| anti-PKM1 | Cat: 15821-1-AP | Proteintech | 1:2000 |
| anti-PKM2 | Cat: 15822-1-AP | Proteintech | 1:2000 |
| anti-PFKM | Cat: 55028-1-AP | Proteintech | 1:2000 |
| anti-4EBP1 | Cat: AF6432 | Affinity | 1:2000 |
| anti-P70S6K | Cat: AF6228 | Affinity | 1:2000 |
| anti-p-P70S6K | Cat: AF3228 | Affinity | 1:2000 |
| anti-p-4EBP1 | Cat: S65 | CST | 1:2000 |
| anti-p-AKT2 | Cat: HA500116 | HUABIO | 1:2000 |
| anti-AKT2 | Cat: HA500091 | HUABIO | 1:2000 |
| anti-TLR5 | Cat: Bs-1197R | Bioss | 1:2000 |
| anti-Myd88 | Cat: Bs-1047R | Bioss | 1:2000 |
| anti-Ub | Cat: 10201-2-AP | Proteintech | 1:2000 |
| anti-β-actin | Cat: 66009-1-Ig | Proteintech | 1:2000 |

**Table S2.** Real-time RT-PCR primers

| Gene name | Direction | Sequence |
| --- | --- | --- |
| *Il6* | Forward  Reverse | 5’-CCGGAGAGGAGACTTCACAG-3’  5’-TCCACGATTTCCCAGAGAAC-3’ |
| *Nos2* | Forward  Reverse | 5’-GTGGTGACAAGCACATTTGG-3’  5’-AAGGCCAAACACAGCATACC-3’ |
| *Arg1* | Forward  Reverse | 5’-GACCTGGCCTTTGTTGATGT-3’  5’-CAGCTCTTCATTGGCTTTCC-3’ |
| *Hk1* | Forward  Reverse | 5’-CCAAAATAGACGAGGCCGTA-3’  5’-TTCAGCAGCTTGACCACATC-3’ |
| *Hk2* | Forward  Reverse | 5’-GAAGATGATCAGCGGGATGT-3’  5’-GCCAGTGGTAAGGAGCTCTG-3’ |
| *Pkm1* | Forward  Reverse | 5’-GCTGTTTGAAGAGCTTGTGC-3'  5’-TTATAAGAGGCCTCCACGCT-3' |
| *Pkm2* | Forward  Reverse | 5’-GTCTGGAGAAACAGCCAAGG-3'  5’-CGGAGTTCCTCGAATAGCTG-3' |
| *Pfkm* | Forward  Reverse | 5’-TGACACAGCACTGAACACCA-3’  5’-AGCCACCCATAGTCTCGATG-3’ |
| *Ccl5* | Forward  Reverse | 5’-ATATGGCTCGGACACCACTC-3’  5’-GCACTTGCTGCTGGTGTAGA-3’ |
| *Cxcl5* | Forward  Reverse | 5’-CGCTAATTTGGAGGTGATCC-3’  5’-TGGATCCAGACAGACCTCCT-3’ |
| *Cxcl9* | Forward  Reverse | 5’-CGCTGTTCTTTTCCTCTTGG-3’  5’-GGAGCATCGTGCATTCCTTA-3’ |
| *Nod2* | Forward  Reverse | 5’-CTCCACTGCCTCTGCCTTAC-3’  5’-GCGAGACTGAGTCAACACCA-3’ |
| *Myd88* | Forward  Reverse | 5’-CTTGATGACCCCCTAGGACA-3’  5’-TCATCACCTGCACAAACTCG-3’ |
| *Tlr9* | Forward  Reverse | 5’-ACTTCGTCCACCTGTCCAAC-3’  5’-TCATGTGGCAAGAGAAGTGC-3’ |
| *Ifi44* | Forward  Reverse | 5’-AGCCCTATGGAGACCTGGTT-3’  5’-TGCCCTTGAACACAGACTTG-3’ |
| *Cxcr2* | Forward  Reverse | 5’-TCTGCTACGGGTTCACACTG-3’  5’-ACAAGGACGACAGCGAAGAT-3’ |
| *Ifng* | Forward  Reverse | 5’-TGAATGTCCAACGCAAAGCA-3'  5’-TCGCTTCCCTGTTTTAGCTG-3' |
| *Tnfa* | Forward  Reverse | 5’-ACGGCATGGATCTCAAAGAC-3’  5’-GTGGGTGAGGAGCACGTAGT-3’ |
| *Ccl2* | Forward  Reverse | 5’-AGGTCCCTGTCATGCTTCTG-3’  5’-CGTTAACTGCATCTGGCTGA-3’ |
| *Il22* | Forward  Reverse | 5’-ATGAGTTTTTCCCTTATGGGGAC-3’  5’-GCTGGAAGTTGGACACCTCAA-3’ |
| *Mmp7* | Forward  Reverse | 5’-CTGCCACTGTCCCAGGA-3’  5’-GGGAGAGTTTTCCAGTCATG-3’ |
| *Reg3α* | Forward  Reverse | 5’-AATGGAGGTGGATGGGAGTG-3’  5’-ACCACGGTTGACAGTAGAGG-3’ |
| *Ang1* | Forward  Reverse | 5’-CACCACTTGTACGCACTCAG-3’  5’-ACTCATCGAAGTGGACAGGC-3’ |
| *Ang4* | Forward  Reverse | 5’-GGTTGTGATTCCTCCAACTCTG-3’  5’-CTGAAGTTTTCTCCATAAGGGC-3’ |
| *Retnlb* | Forward  Reverse | 5’-CATCCTCGTCTCCCTTCTCC-3’  5’-GCAGGAGATCGTCTTAGGCT-3’ |
| *Ltln1* | Forward  Reverse | 5’-GAGCACACAAAGGCACAAGA-3’  5’-ACTTCCCACGCATGTTGTTC-3’ |
| *Actb* | Forward  Reverse | 5’-GCTACAGCTTCACCACCACA-3’  5’-TCTCCAGGGAGGAAGAGGAT-3’ |

**Table S3.** Fecal DNA PCR primers

| Gene name | Direction | Sequence |
| --- | --- | --- |
| B.*fragilis* | Forward  Reverse | 5’-TGATTCCGCCATGGTTTCATT-3’  5’-CGACCCATAGAGCCTTCATC-3’ |
| *Parasutterella* | Forward  Reverse | 5’-GGAAGTACGGTCGCAAGA-3’  5’-TGTCAAGGGTTGGGTAAGACA-3’ |
| *Butyricicoccus* | Forward  Reverse | 5’-ACCTGAAGAATAAGCTCC-3’  5’-GATAACGTTGCTCCCTACGT-3’ |
| *Bifidobacterium* | Forward  Reverse | 5’-CTCCTGGAAACGGGTGG-3’  5’-GGTGTTCTTCCCGATATCTACA-3’ |
| *16S* | Forward  Reverse | 5’-ACTCCTACGGGAGGCAGCAG-3’  5’-ATTACCGCGGCTGCTGG-3’ |
