# Supplementary material for: Blockade of C5aR1 resets M1 via gut microbiota-mediated PFKM stabilization in a TLR5-dependent manner
Source: Cell Death Dis. 2024 Feb 8;15(2):120. doi: 10.1038/s41419-024-06500-4 (PMC10853248; doi:10.1038/s41419-024-06500-4)
Supplement: Supplementary file 2 — Original Data File [file 41419_2024_6500_MOESM2_ESM.pdf]

Figure 2C

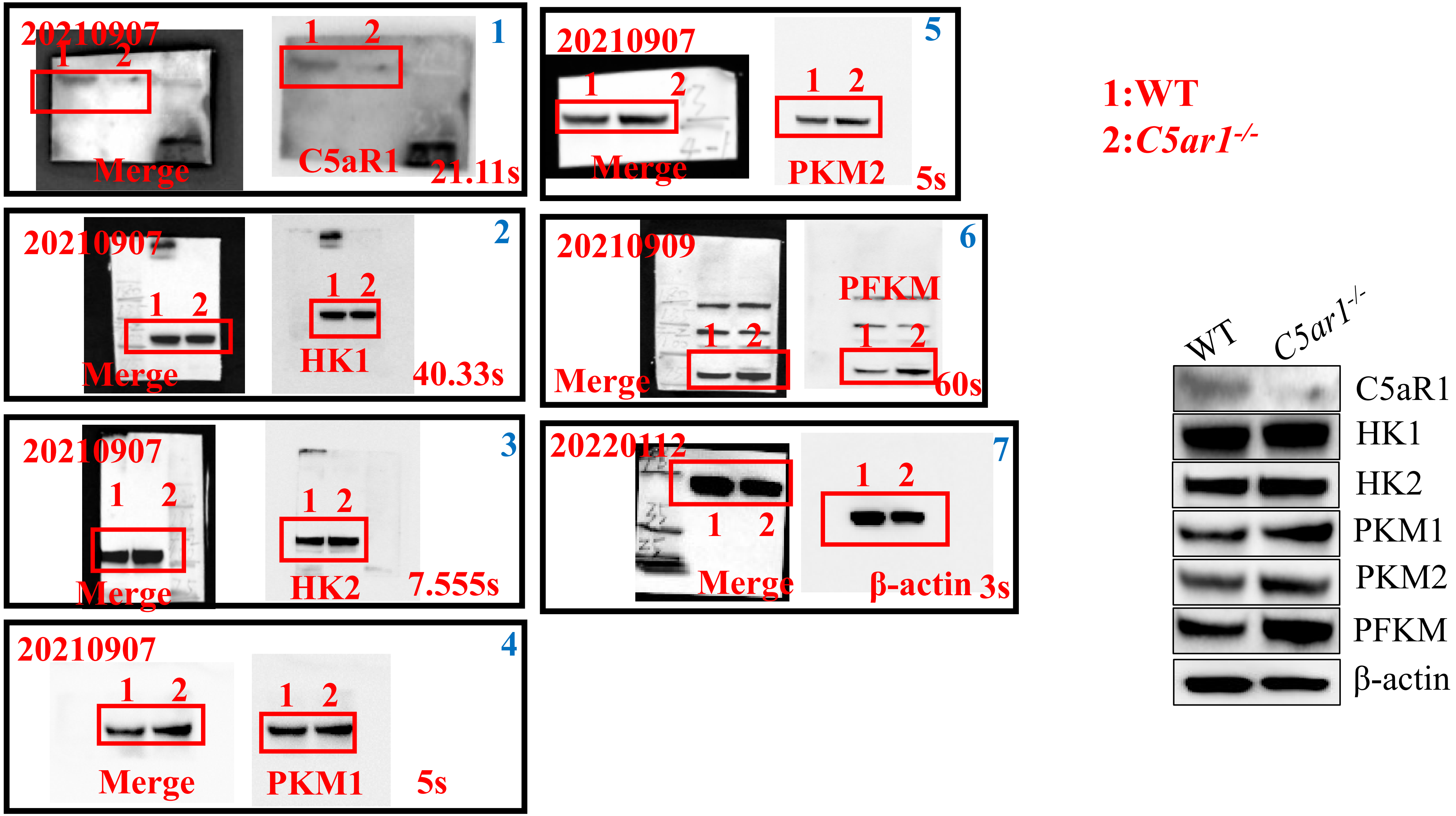

1
20210907
1 2
Merge
C5aR1
21.11s
2
20210907
1 2
Merge
HK1
40.33s
3
20210907
1 2
Merge
HK2
7.555s
4
20210907
1 2
Merge
PKM1
5s
5
20210907
1 2
Merge
PKM2
5s
6
20210909
1 2
Merge
PFKM
60s
7
20220112
1 2
Merge
β-actin 3s
1:WT
2:*C5ar1*-/-
WT
C5ar1-/-
C5aR1
HK1
HK2
PKM1
PKM2
PFKM
β-actin

Figure 2D

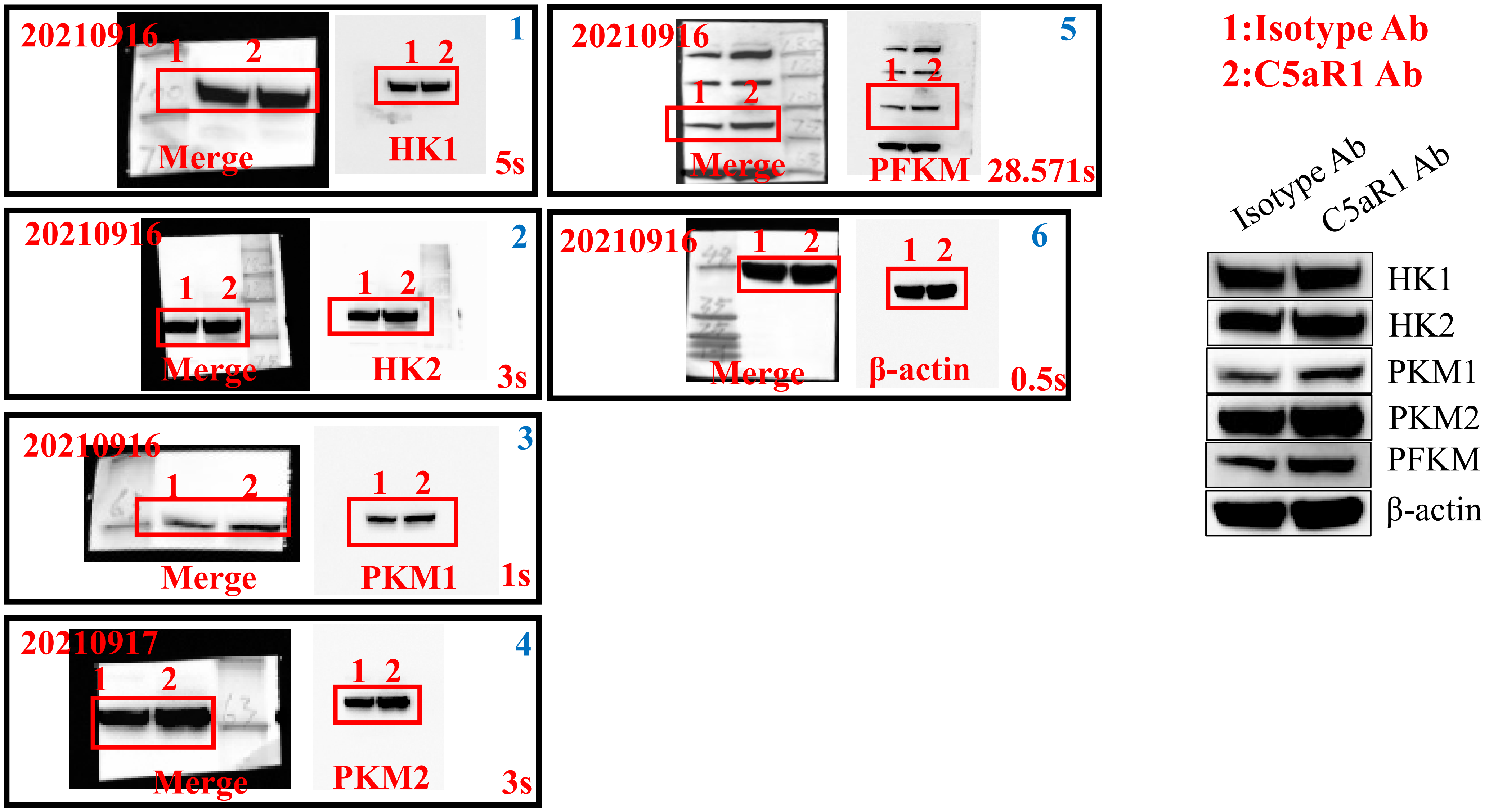

20210916
1 2
Merge
1 2
HK1
1
5s
20210916
1 2
Merge
1 2
PFKM
5
28.571s
20210916
1 2
Merge
1 2
HK2
2
3s
20210916
1 2
Merge
1 2
β-actin
6
0.5s
20210916
1 2
Merge
1 2
PKM1
3
1s
20210917
1 2
Merge
1 2
PKM2
4
3s
1:Isotype Ab
2:C5aR1 Ab
Isotype Ab
C5aR1 Ab
HK1
HK2
PKM1
PKM2
PFKM
β-actin

Figure 2E

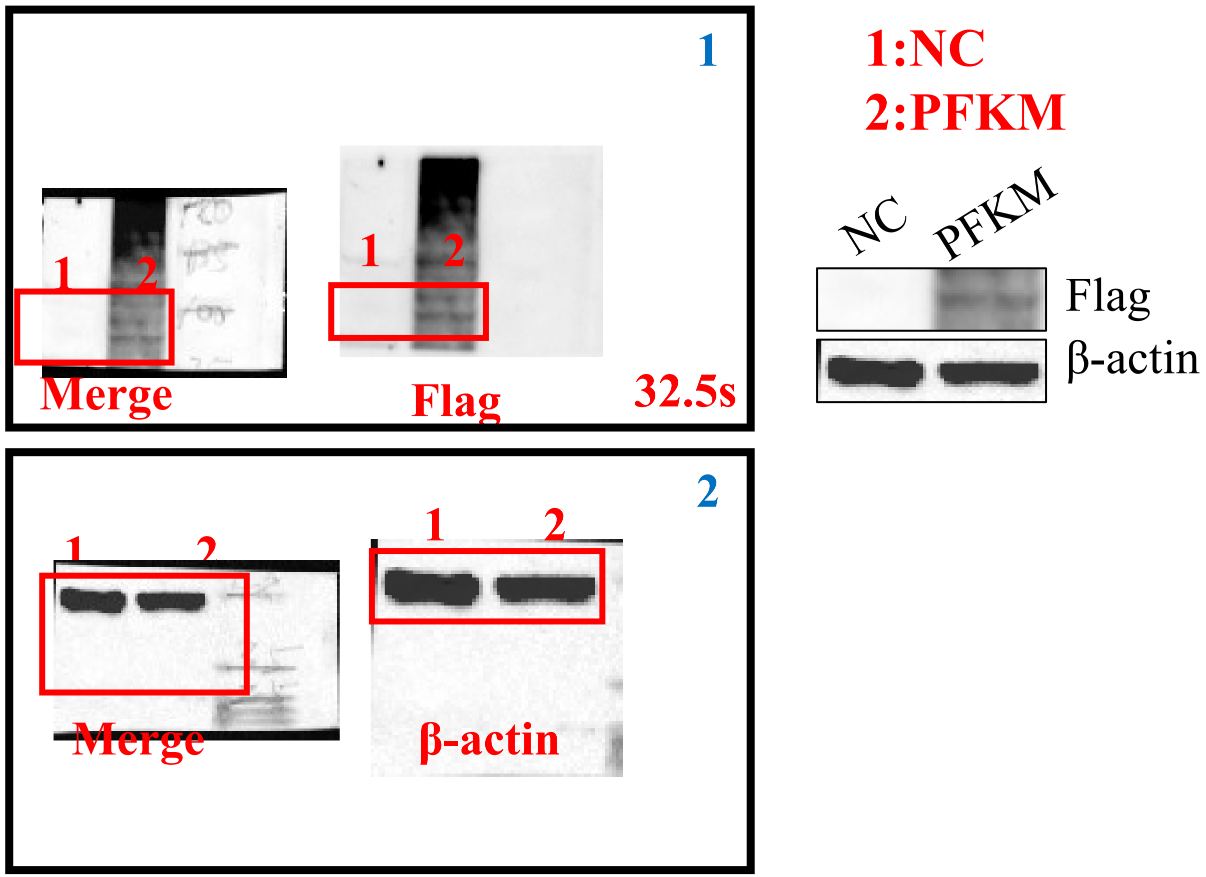
1
1 2
Merge
1 2
Flag
32.5s
2
1 2
Merge
1 2
β-actin
1:NC
2:PFKM
NC
PFKM
Flag
β-actin

Figure 2G

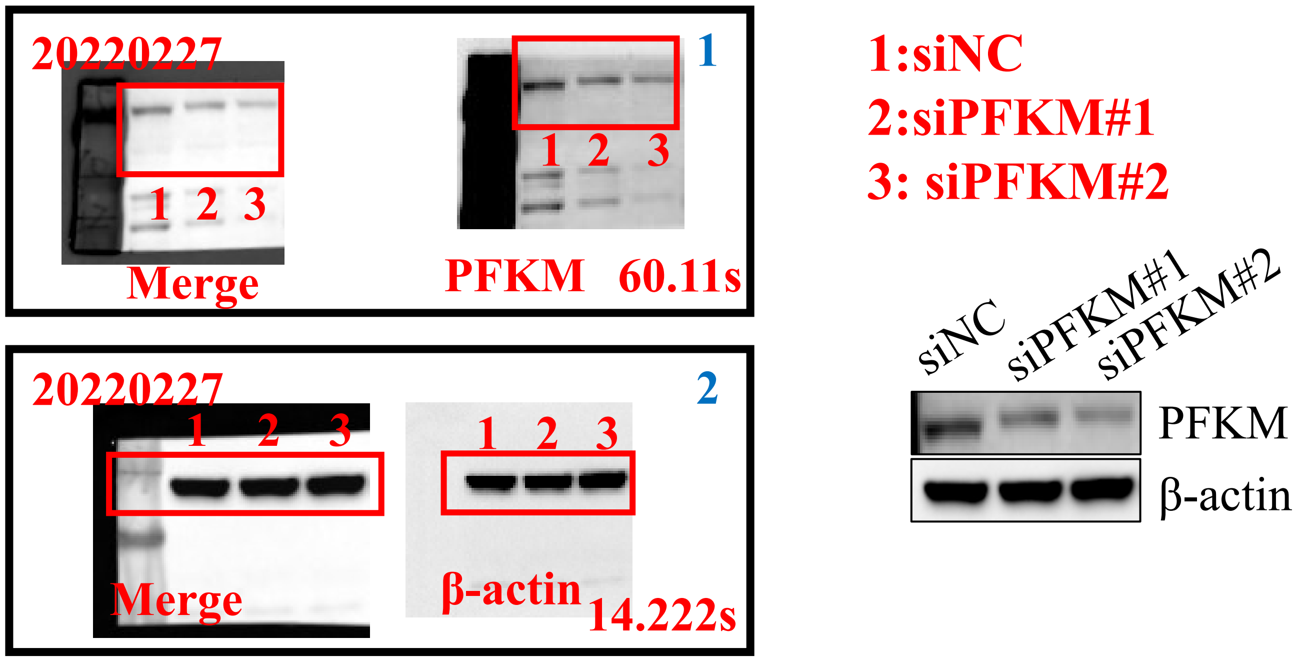
20220227
1 2 3
Merge
1
1 2 3
PFKM 60.11s
20220227
1 2 3
Merge
2
1 2 3
β-actin
14.222s
1:siNC
2:siPFKM#1
3: siPFKM#2
siNC
siPFKM#1
siPFKM#2
PFKM
β-actin

Figure 2I

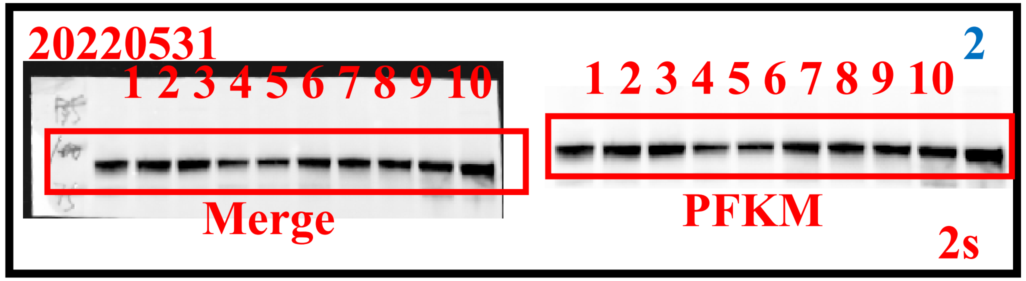

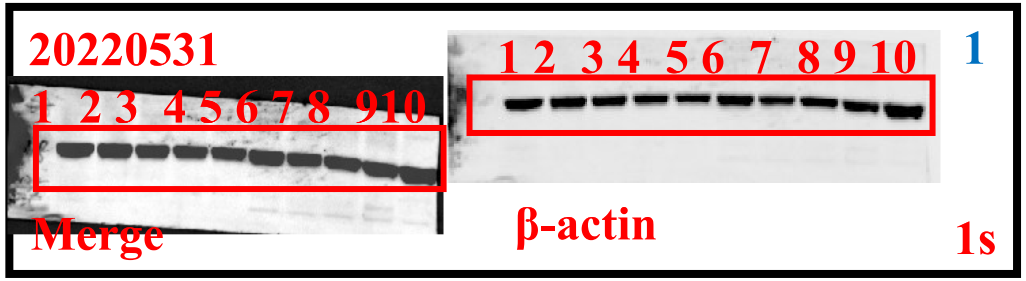

1:Isotype Ab(0h)
2:Isotype Ab(1h)
3:Isotype Ab(2h)
4:Isotype Ab(4h)
5:Isotype Ab(8h)
6:C5aR1 Ab(0h)
7:C5aR1 Ab(1h)
8:C5aR1 Ab(2h)
9:C5aR1 Ab(4h)
10:C5aR1 Ab(8h)

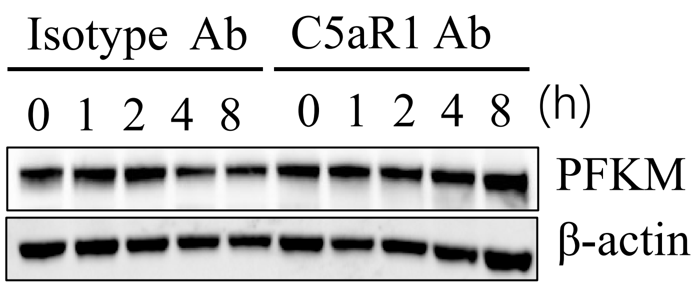

Figure 2J

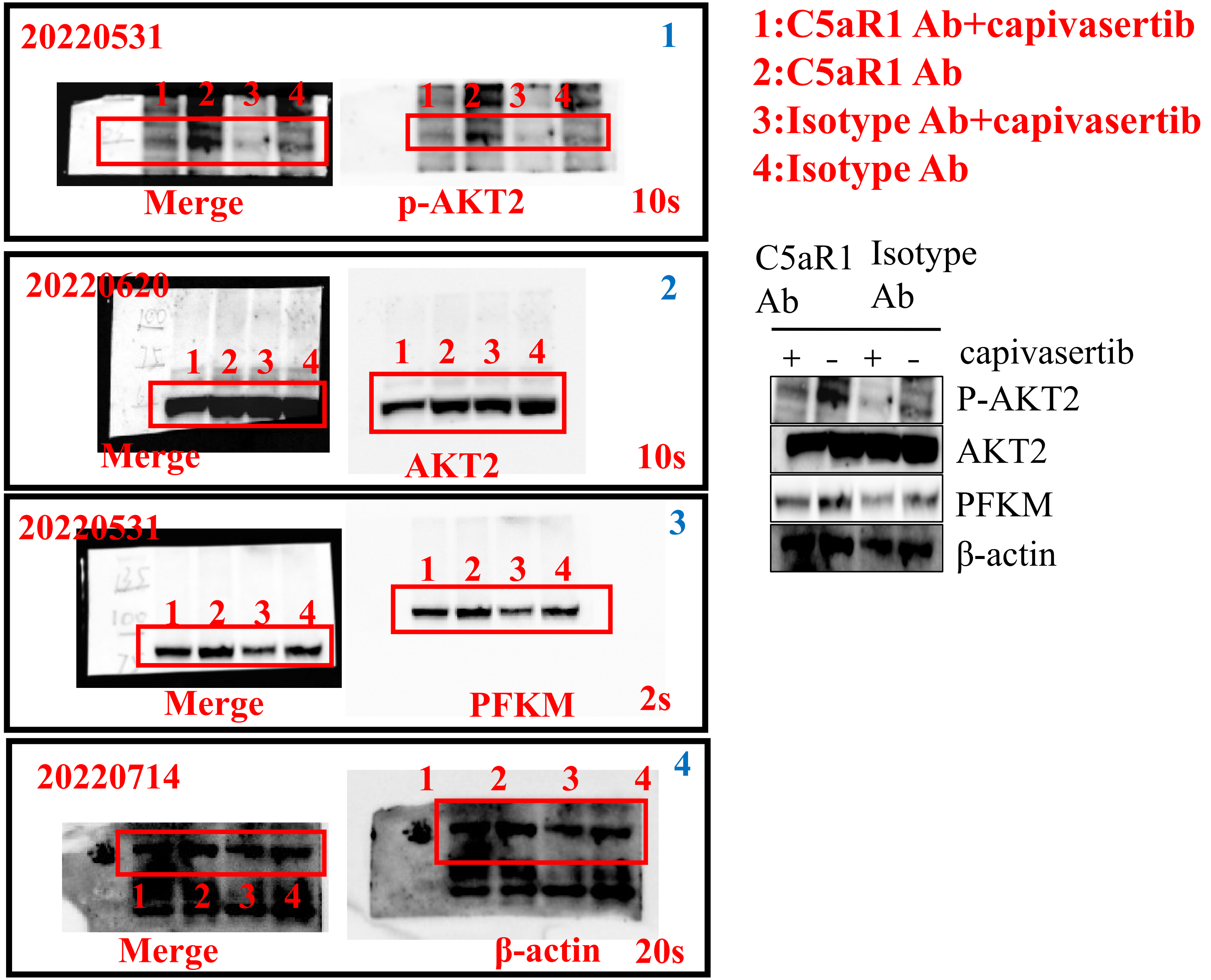

20220531
1
1 2 3 4
Merge
p-AKT2
10s
20220620
2
1 2 3 4
Merge
AKT2
10s
20220531
3
1 2 3 4
Merge
PFKM
2s
20220714
4
1 2 3 4
Merge
β-actin
20s
1:C5aR1 Ab+capivasertib
2:C5aR1 Ab
3:Isotype Ab+capivasertib
4:Isotype Ab
C5aR1 Ab
Isotype Ab
+ - + -
capivasertib
P-AKT2
AKT2
PFKM
β-actin

Figure 2K

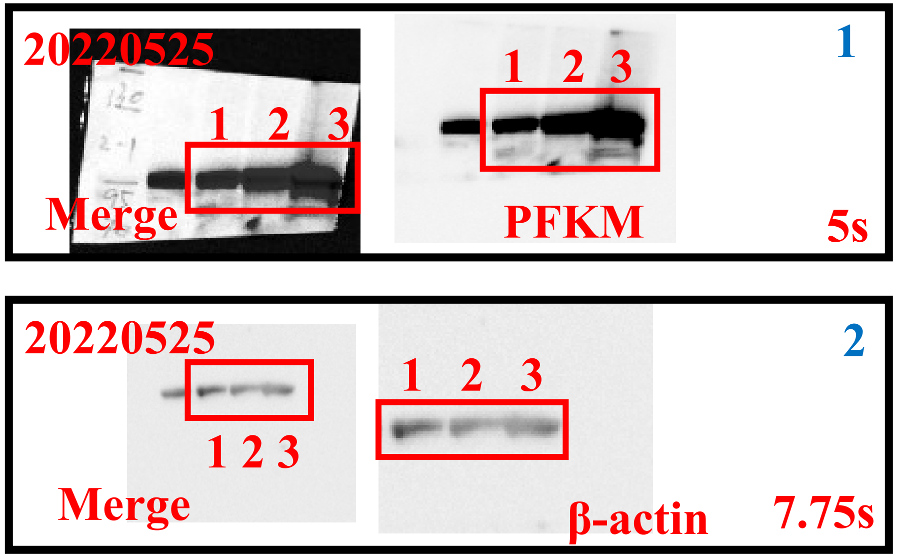

1:Isotype Ab
2:C5aR1 Ab
3:C5aR1
4:Ab+MG132

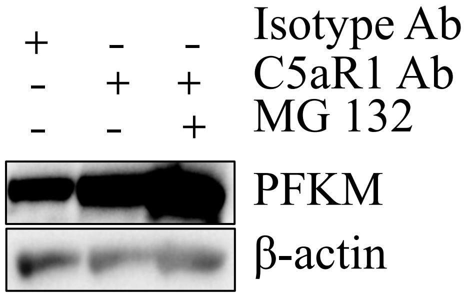

Figure 2L

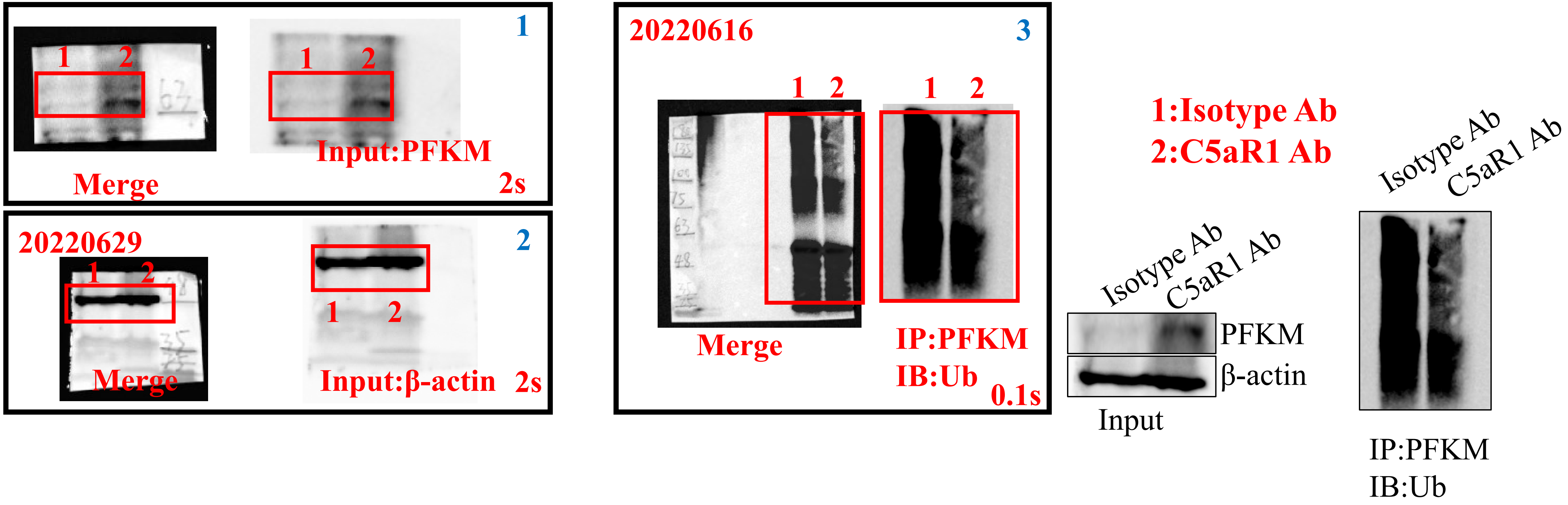

1
1 2
Merge
Input:PFKM
2s
20220629
1 2
Merge
2
1 2
Input:β-actin
2s
20220616
3
1 2
Merge
IP:PFKM
IB:Ub
0.1s
1:Isotype Ab
2:C5aR1 Ab
Isotype Ab
C5aR1 Ab
PFKM
β-actin
Input
IP:PFKM
IB:Ub

Figure 3H

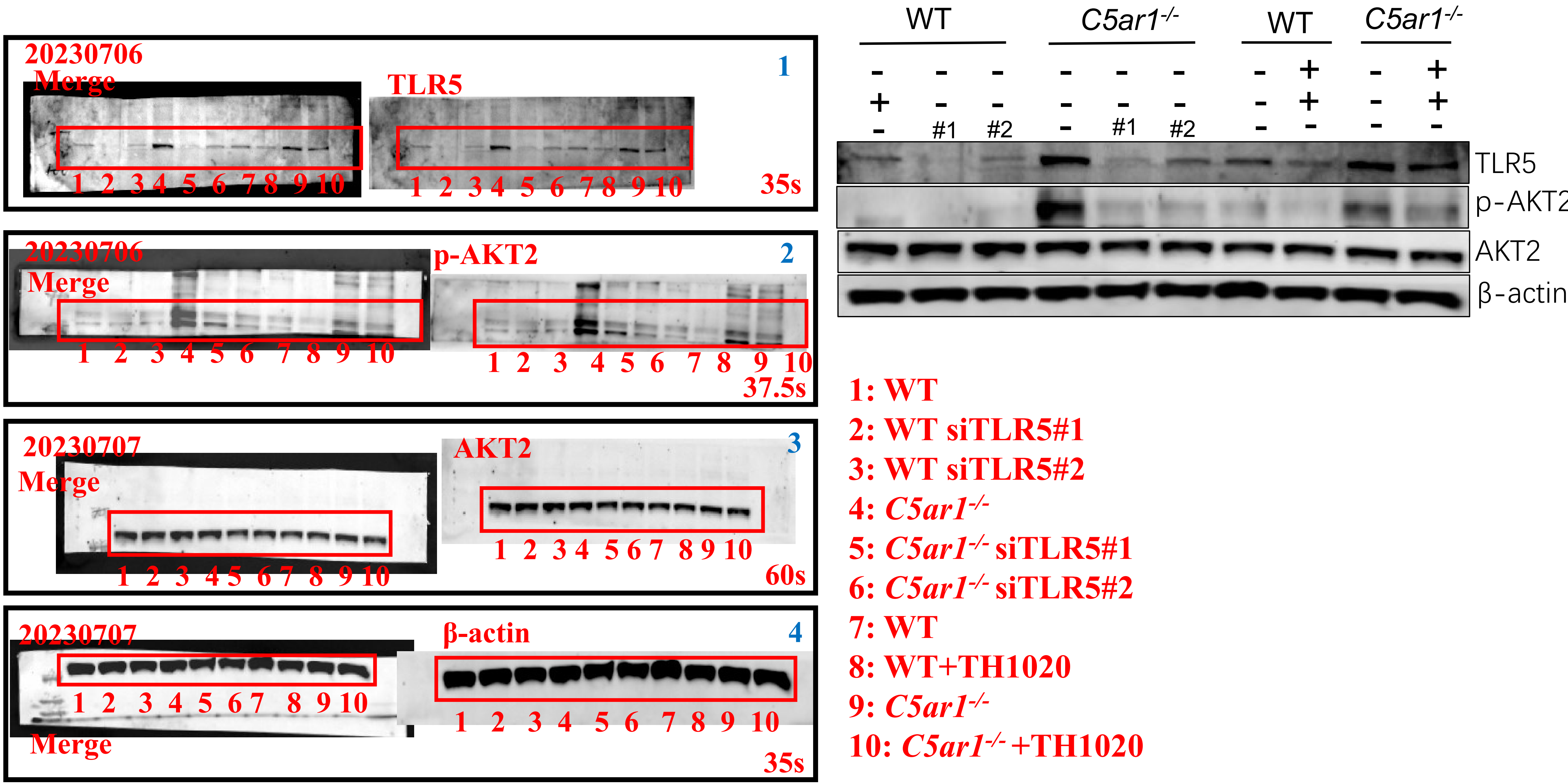
20230706
Merge
TLR5
1
35s
20230706
Merge
p-AKT2
2
37.5s
20230707
Merge
AKT2
3
60s
20230707
Merge
β-actin
4
35s
WT
C5ar1-/-
WT
C5ar1-/-
#1
#2
#1
#2
TLR5
p-AKT2
AKT2
β-actin
1: WT
2: WT siTLR5#1
3: WT siTLR5#2
4: C5ar1-/-
5: C5ar1-/- siTLR5#1
6: C5ar1-/- siTLR5#2
7: WT
8: WT+TH1020
9: C5ar1-/-
10: C5ar1-/- +TH1020

Figure 3I-1

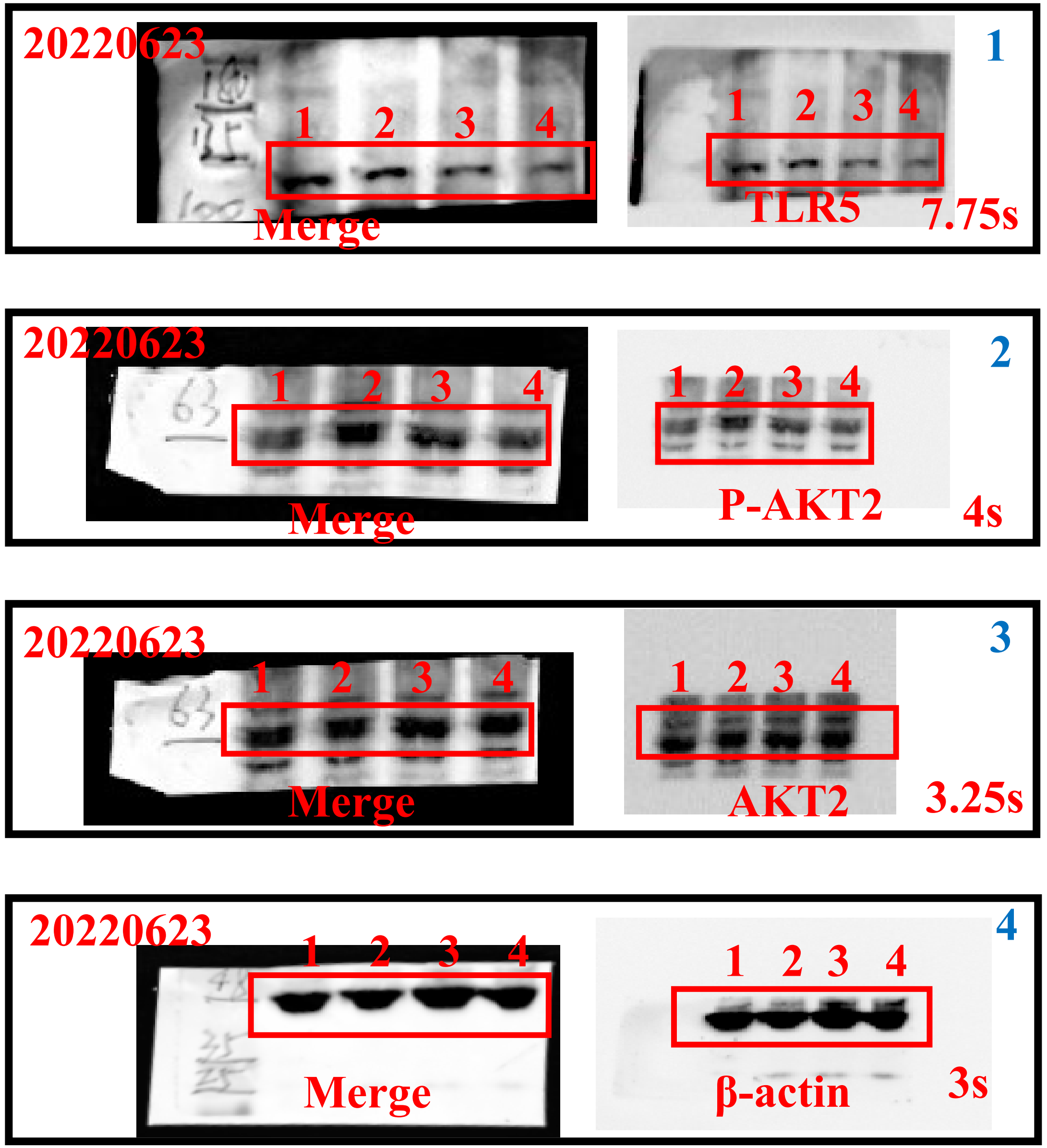

1:isotype Ab
2:C5aR1 Ab+siNC
3:C5aR1 Ab+siTLR5#1
4:C5aR1 Ab+siTLR5#2

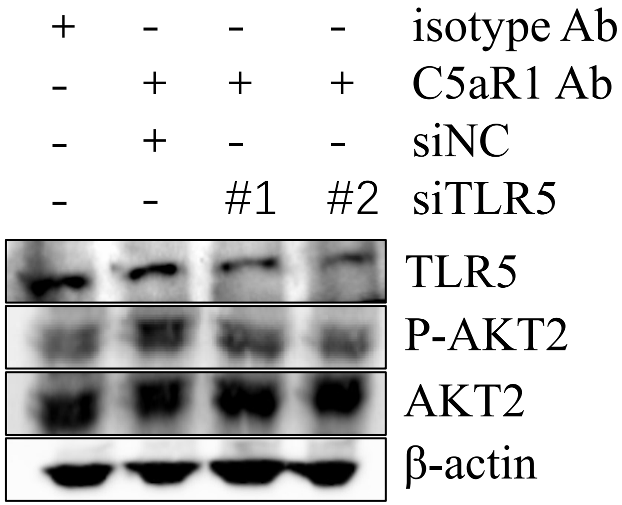

Figure 3I-2

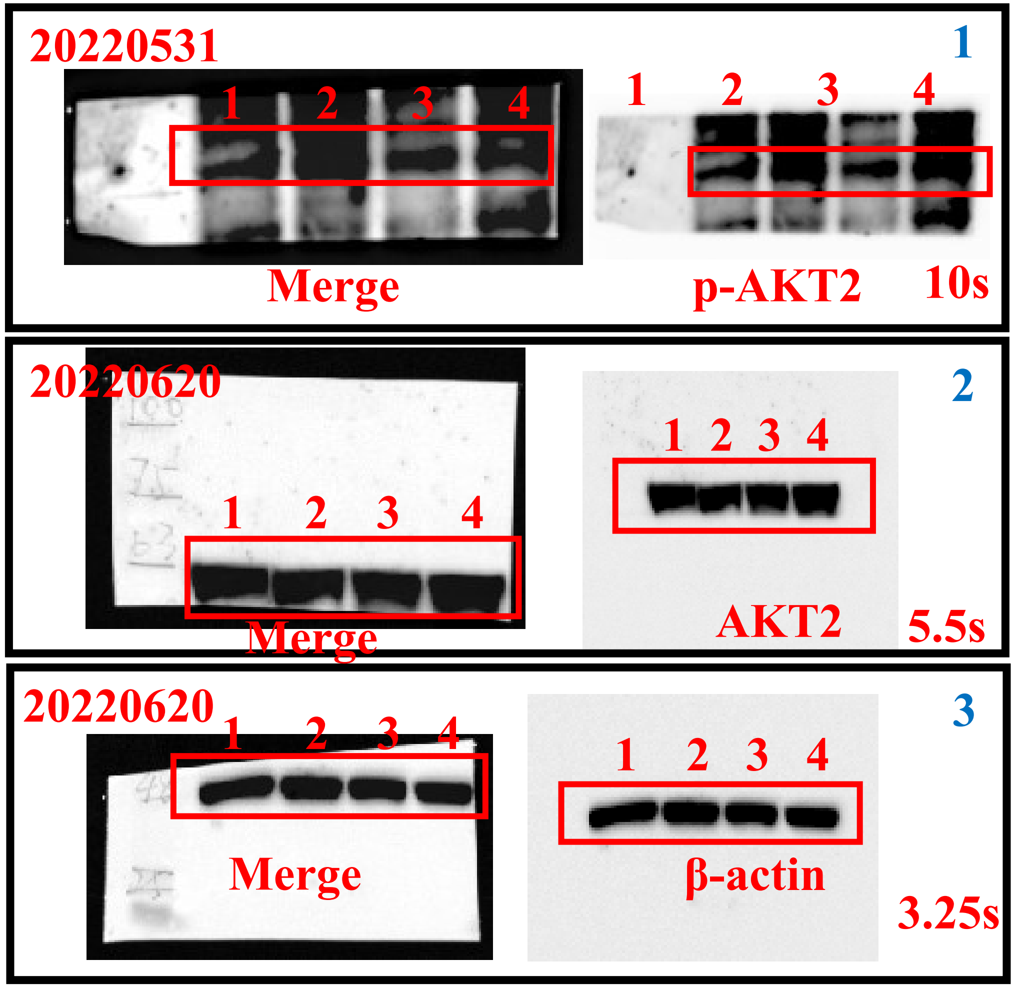

1:C5aR1 Ab+TH1020
2:C5aR1 Ab
3:Isotype Ab+TH1020
4:Isotype Ab

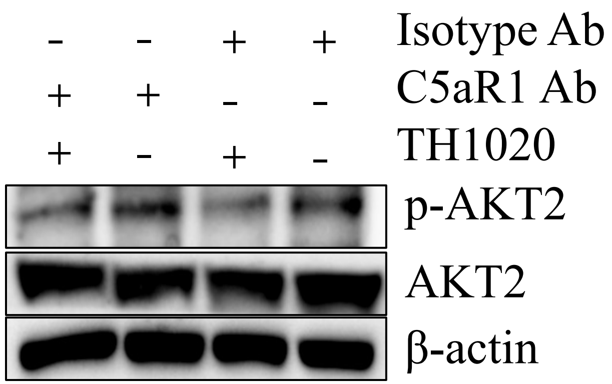

Figure 3J

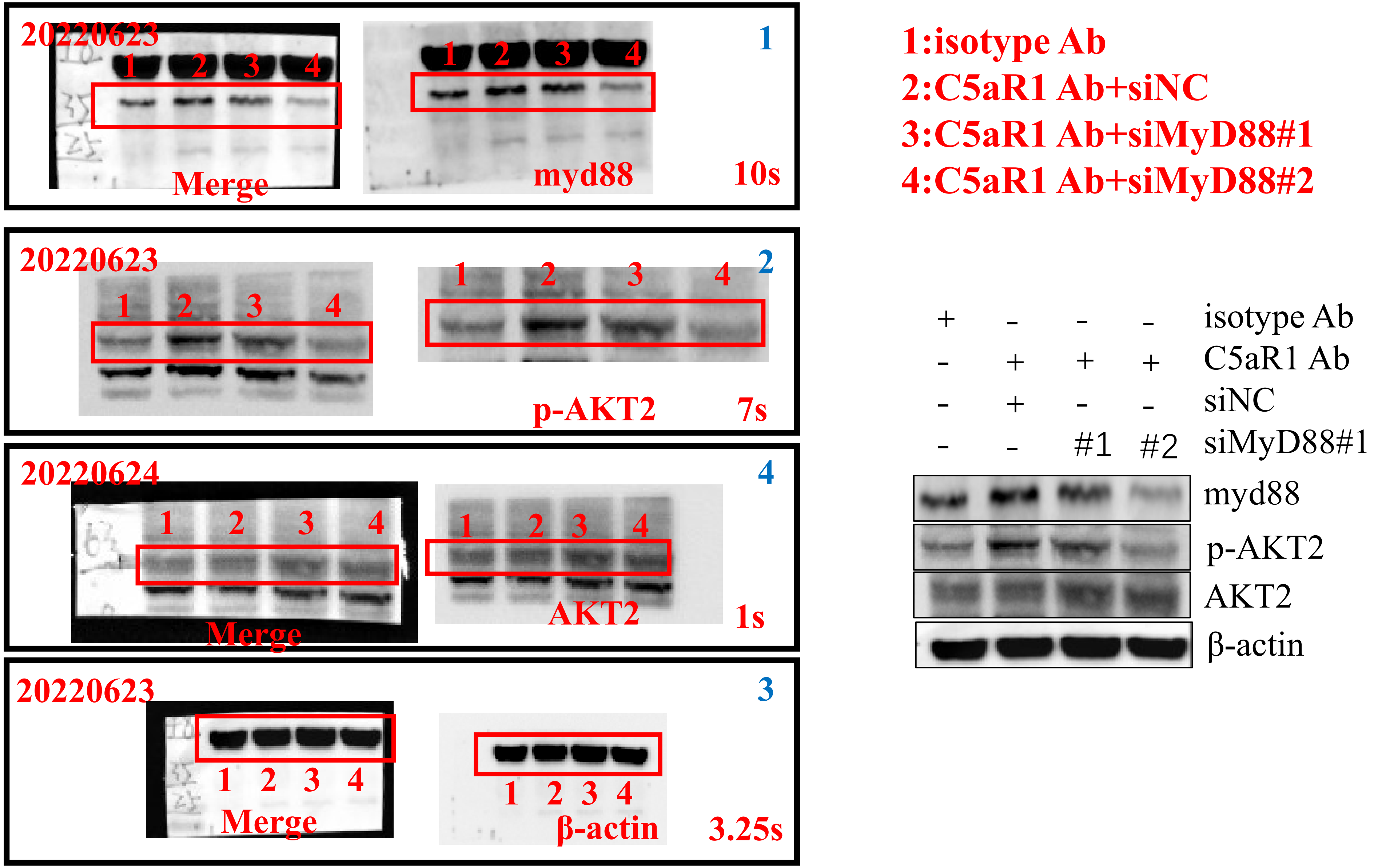
1:isotype Ab
2:C5aR1 Ab+siNC
3:C5aR1 Ab+siMyD88#1
4:C5aR1 Ab+siMyD88#2
20220623
Merge
myd88
10s
1
20220623
p-AKT2
7s
2
20220624
Merge
AKT2
1s
4
20220623
Merge
β-actin
3.25s
3
isotype Ab
C5aR1 Ab
siNC
siMyD88#1
#1
#2
myd88
p-AKT2
AKT2
β-actin

Figure S1I

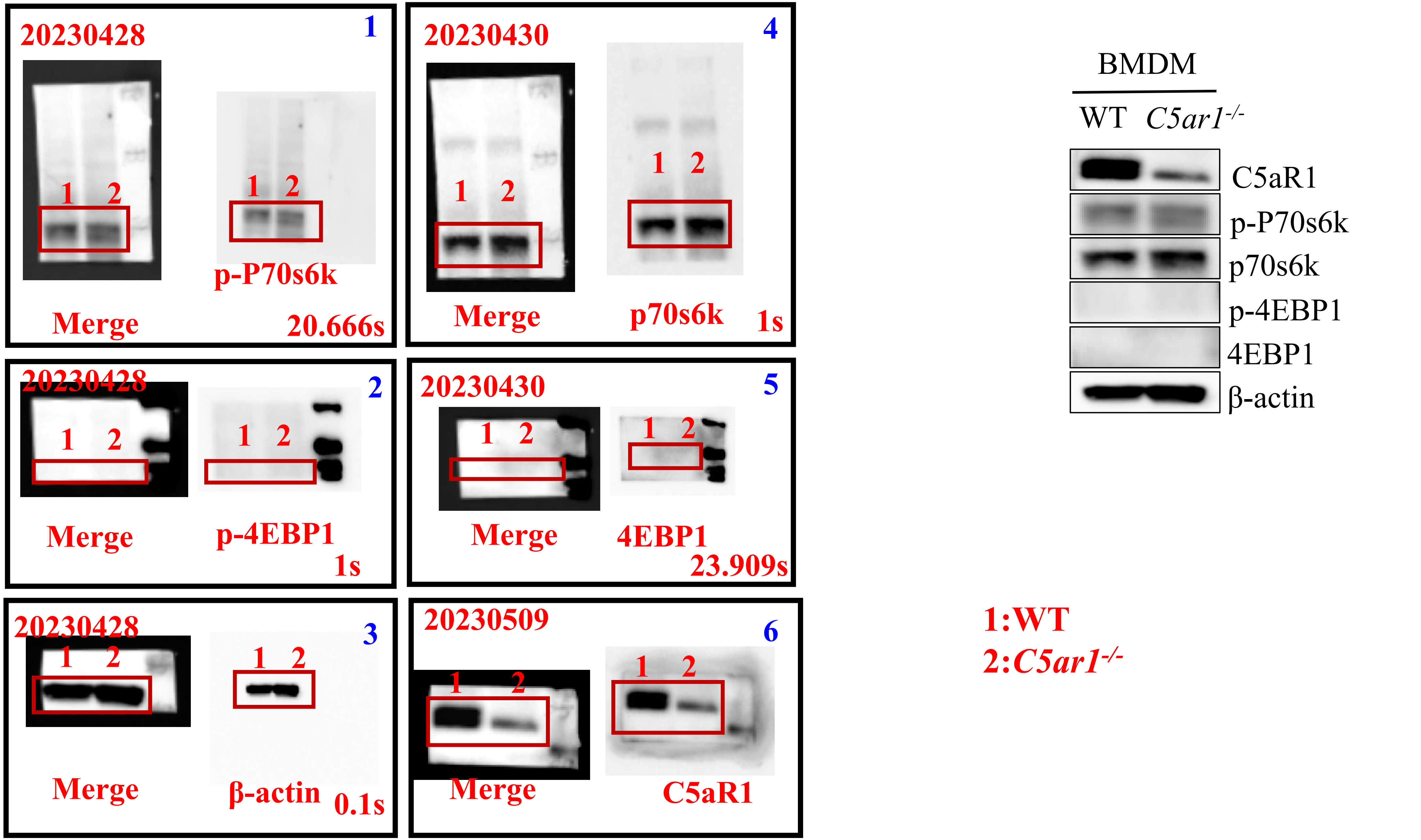

20230428
1
1 2
1 2
p-P70s6k
Merge
20.666s
20230430
4
1 2
1 2
Merge
p70s6k
1s
20230428
2
1 2
1 2
Merge
p-4EBP1
1s
20230430
5
1 2
1 2
Merge
4EBP1
23.909s
20230428
3
1 2
1 2
Merge
β-actin
0.1s
20230509
6
1 2
1 2
Merge
C5aR1
BMDM
WT
C5ar1-/-
C5aR1
p-P70s6k
p70s6k
p-4EBP1
4EBP1
β-actin
1:WT
2:C5ar1-/-

Figure S2D

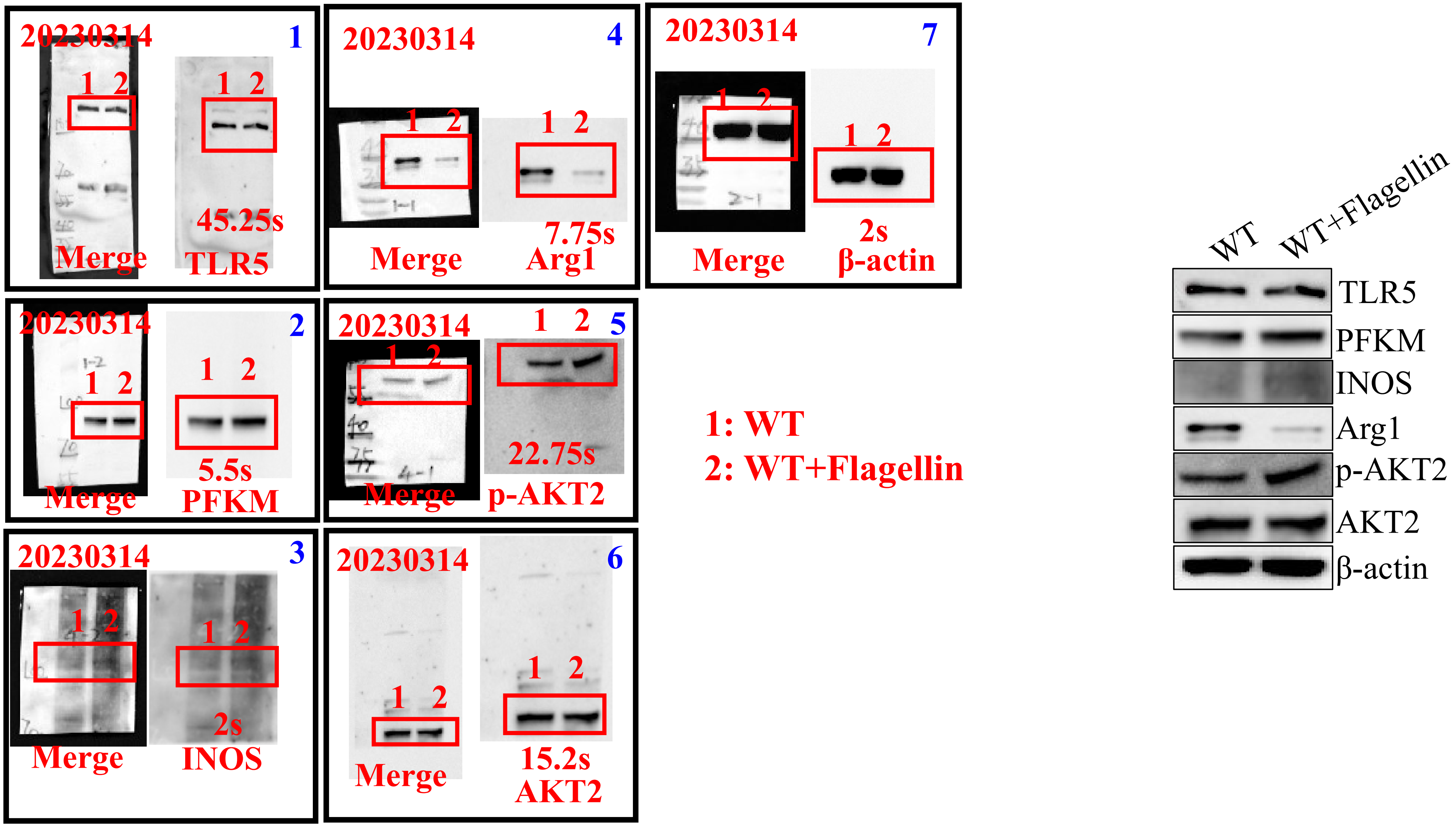
20230314
1
1 2
1 2
45.25s
Merge
TLR5
20230314
4
1 2
1 2
7.75s
Merge
Arg1
20230314
7
1 2
1 2
2s
Merge
β-actin
20230314
2
1 2
1 2
5.5s
Merge
PFKM
20230314
1 2
5
1 2
22.75s
Merge
p-AKT2
20230314
3
1 2
1 2
2s
Merge
INOS
20230314
6
1 2
1 2
15.2s
Merge
AKT2
1: WT
2: WT+Flagellin
WT
WT+Flagellin
TLR5
PFKM
INOS
Arg1
p-AKT2
AKT2
β-actin

Figure S2E

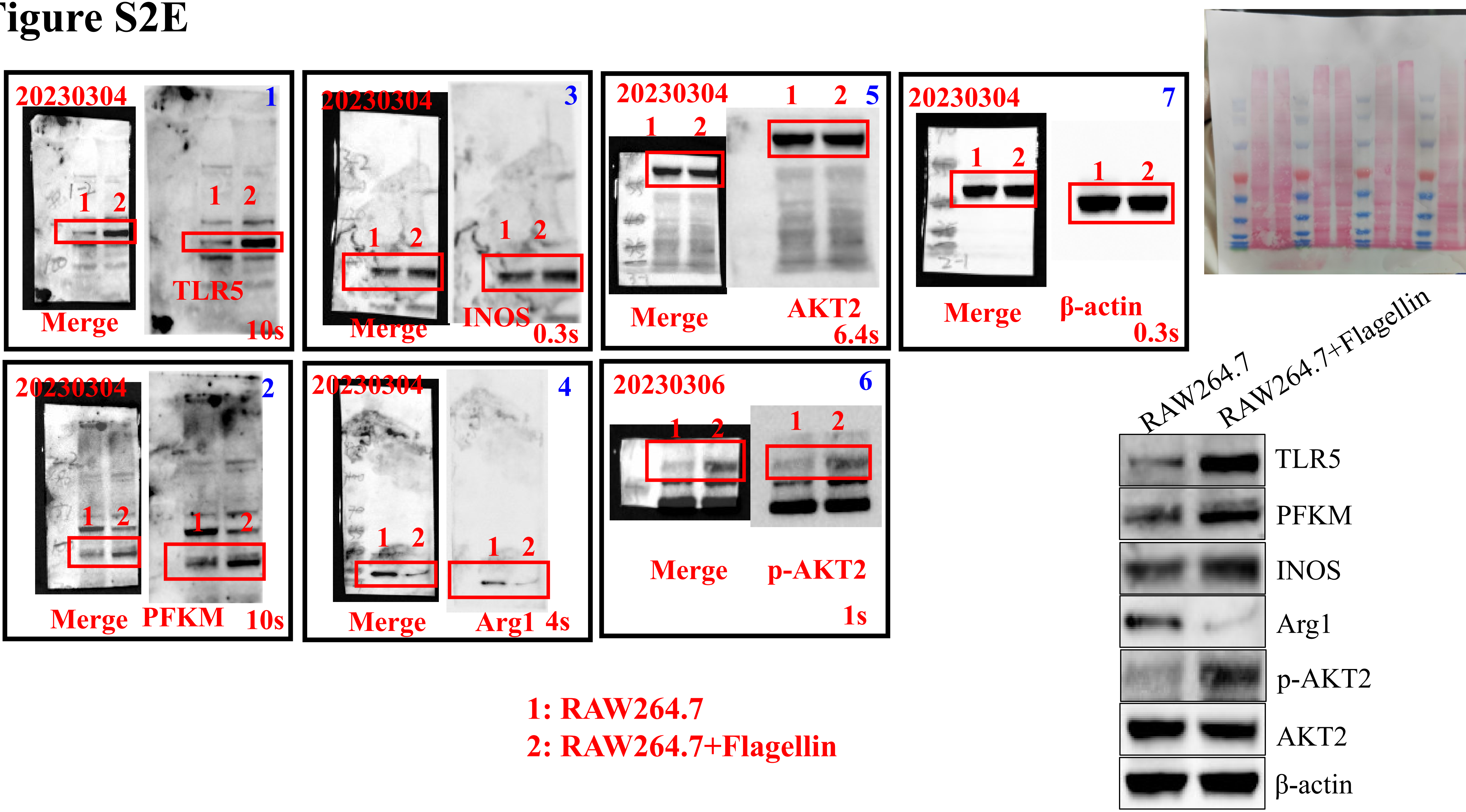

20230304
1
TLR5
10s
Merge
1 2
20230304
3
INOS
0.3s
Merge
20230304
5
AKT2
6.4s
Merge
20230304
7
β-actin
0.3s
Merge
20230304
2
PFKM
10s
Merge
20230304
4
Arg1 4s
Merge
20230306
6
p-AKT2
1s
Merge
RAW264.7
RAW264.7+Flagellin
TLR5
PFKM
INOS
Arg1
p-AKT2
AKT2
β-actin
1: RAW264.7
2: RAW264.7+Flagellin
